# Supplementary material for: A quasi-integral controller for adaptation of genetic modules to variable ribosome demand
Source: Nat Commun. 2018 Dec 21;9:5415. doi: 10.1038/s41467-018-07899-z (PMC6303309; doi:10.1038/s41467-018-07899-z)
Supplement: Supplementary file 1 — Supplementary Information [file 41467_2018_7899_MOESM1_ESM.pdf]

A quasi-integral controller for adaptation of genetic modules to variable ribosome demand

Supplementary Information

Hsin-Ho Huang[1†], Yili Qian[1†] and Domitilla Del Vecchio[1,2,*]

[1] Department of Mechanical Engineering, Massachusetts Institute of Technology, 77 Massachusetts Avenue, Cambridge, MA02139, USA
[2] Synthetic Biology Center, Massachusetts Institute of Technology, 500 Technology Square, Cambridge, MA02139, USA
[†] These authors contributed equally to this work.

Plasmid	Fragment	Forward	Reverse	Template DNA	Size (bp)
pHH01_15	1	P137	HH03_R3	pHH01_15_436_Ac #2	1673
	2	HH03_F3	P88	pHH01_15_436_Ac #2	1148
	3	P87	P138	pHH29_32_lib62 #1	801
pHH01_22	1	P137	HH03_R3	pHH01_22_4450_Ac #1	1589
	2	HH03_F3	P88	pHH01_15_436_Ac #2	1148
	3	P87	P138	pHH29_32_lib62 #1	801
pHH01_32	1	P137	HH03_R3	pHH01_32_1122_Ac #3	1637
	2	HH03_F3	P88	pHH01_15_436_Ac #2	1148
	3	P87	P138	pHH29_32_lib62 #1	801
pHH02_15UP436	1	HH02_UP_F	GFP_R	pVRb15_up436	954
	2	ColE1_F	HH02_UP_R	pZE21_GFP_ColE2_Cm	1723
pHH02_22UP1147	1	HH02_UP_F	GFP_R	pVRb22_up1147	954
	2	ColE1_F	HH02_UP_R	pZE21_GFP_ColE2_Cm	1723
pHH02_32_1122	1	HH02_32_F	GFP_R	pVRb32_1122	954
	2	ColE1_F	HH02_32_R	pZE21_GFP_ColE2_Cm	1723
pHH03C_32	1	P89	HH01_32_R	pHH03b_Ac #10	1730
	2	HH01_32_F	P88	pHH03b_Ac #10	1872
	3	P87	P90	pHH29_32_lib62 #1	789
pHH04C_Sp_coD	1	HHC1_F7	HH06_R4	pHH04_32_sRNA-A_Sp #1	775
	2	HH06_F4	HH06_R3	pHH04_32_RZ491A_Sp#3	1419
pHH04_32_sRNA-A_Sp_coD	1	HH06_F3	HH06_R4	pHH04_32_sRNA-A_Sp #1	941
	2	HH06_F4	HH06_R3	pHH04_32_RZ491A_Sp#3	1419
pHH29_32_HG02	1	P41	HH13_R3	pHH29_32_lib62-5 #2	1911
	2	HH13_F3	P180	pHH29_32_lib62-5 #2	1921
	3	P179	P42	pHH29_32_lib62-5 #2	1610
pHH29_32_HG18	1	P41	HH13_R3	pHH29_32_lib62-5 #2	1911
	2	HH13_F3	P180	pHH29_32_lib62-5 #2	1921
	3	P179	P201	pHH29_32_lib62-5 #2	808
	4	P200	P42	pHH29_32_lib62-5 #2	831
pHH29_32_HG19	1	P41	HH13_R3	pHH29_32_lib62-5 #2	1911
	2	HH13_F3	P180	pHH29_32_lib62-5 #2	1921
	3	P179	P28	pHH29_32_lib62-5 #2	811
	4	P27	P42	pHH29_32_lib62-5 #2	825
pHH29_32_HG27	1	P41	HH13_R3	pHH29_32_HG05 #2	1911
	2	HH13_F3	P211	pHH29_32_HG05 #2	1942
	3	P210	P42	pHH29_32_HG05 #2	1613
pHH29_32_HG35	1	P41	HH13_R3	pHH29_32_HG27 #2	1911
	2	HH13_F3	P88	pHH29_32_HG27 #2	1942
	3	P87	P201	pHH29_32_HG27 #2	1605
	4	P200	P42	pHH29_32_HG27 #2	831
pHH29_32_HG36	1	P41	HH13_R3	pHH29_32_HG27 #2	1911
	2	HH13_F3	P88	pHH29_32_HG27 #2	1942
	3	P87	P28	pHH29_32_HG27 #2	1608
	4	P27	P42	pHH29_32_HG27 #2	825

Supplementary Table 1: The list of plasmids assembled by Gibson assembly. Each fragment was prepared by the indicated forward and reverse primers and the template DNA. DNA sequences are available on Addgene (#120890-120901). The annotated plasmid maps are provided in Supplementary Figure 1-3.

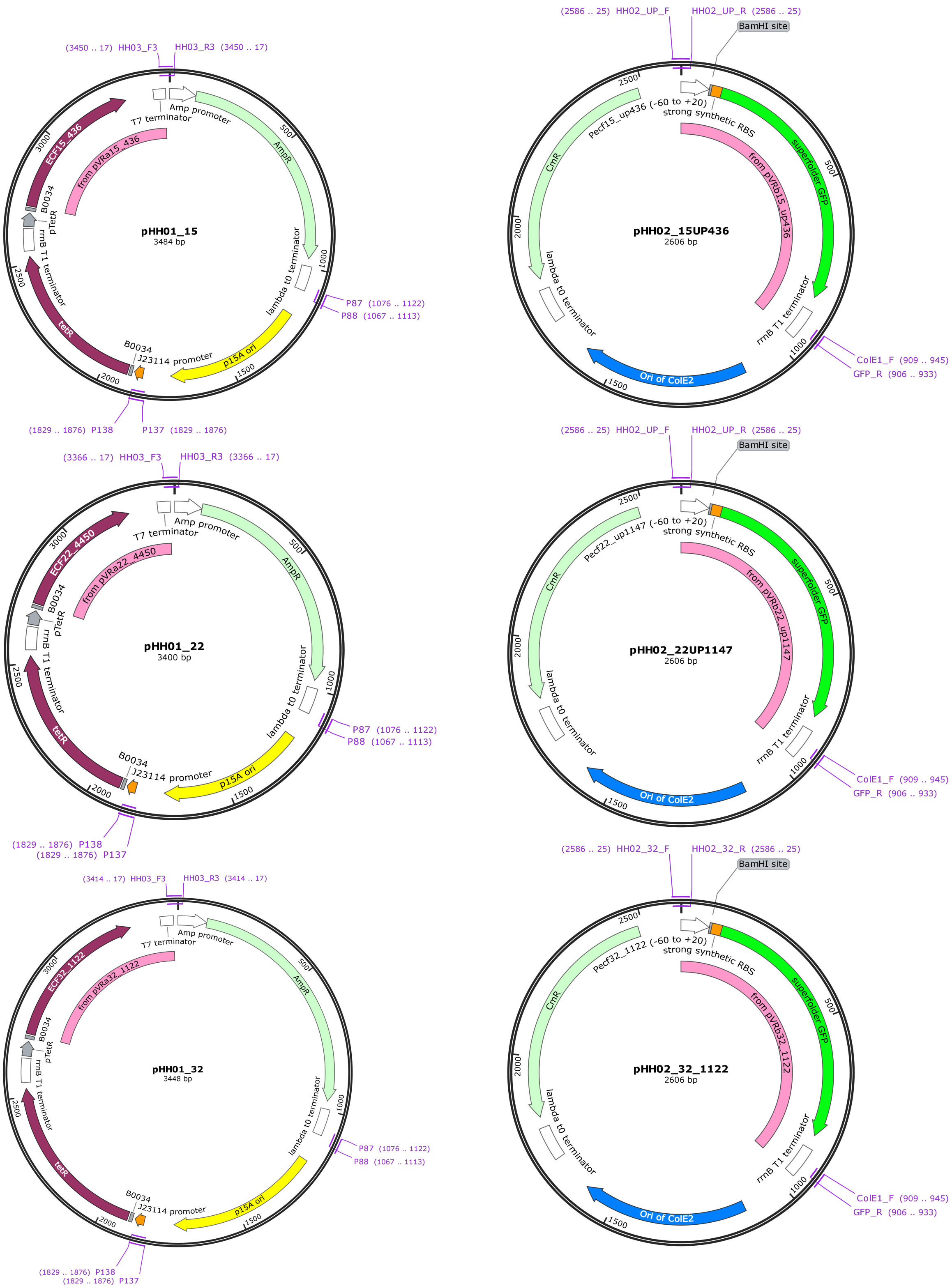

Supplementary Figure 1: The maps of pHH01 and pHH02 plasmids. The assembled fragments can be found in Supplementary Table 1. The maps are prepared by the SnapGene software.

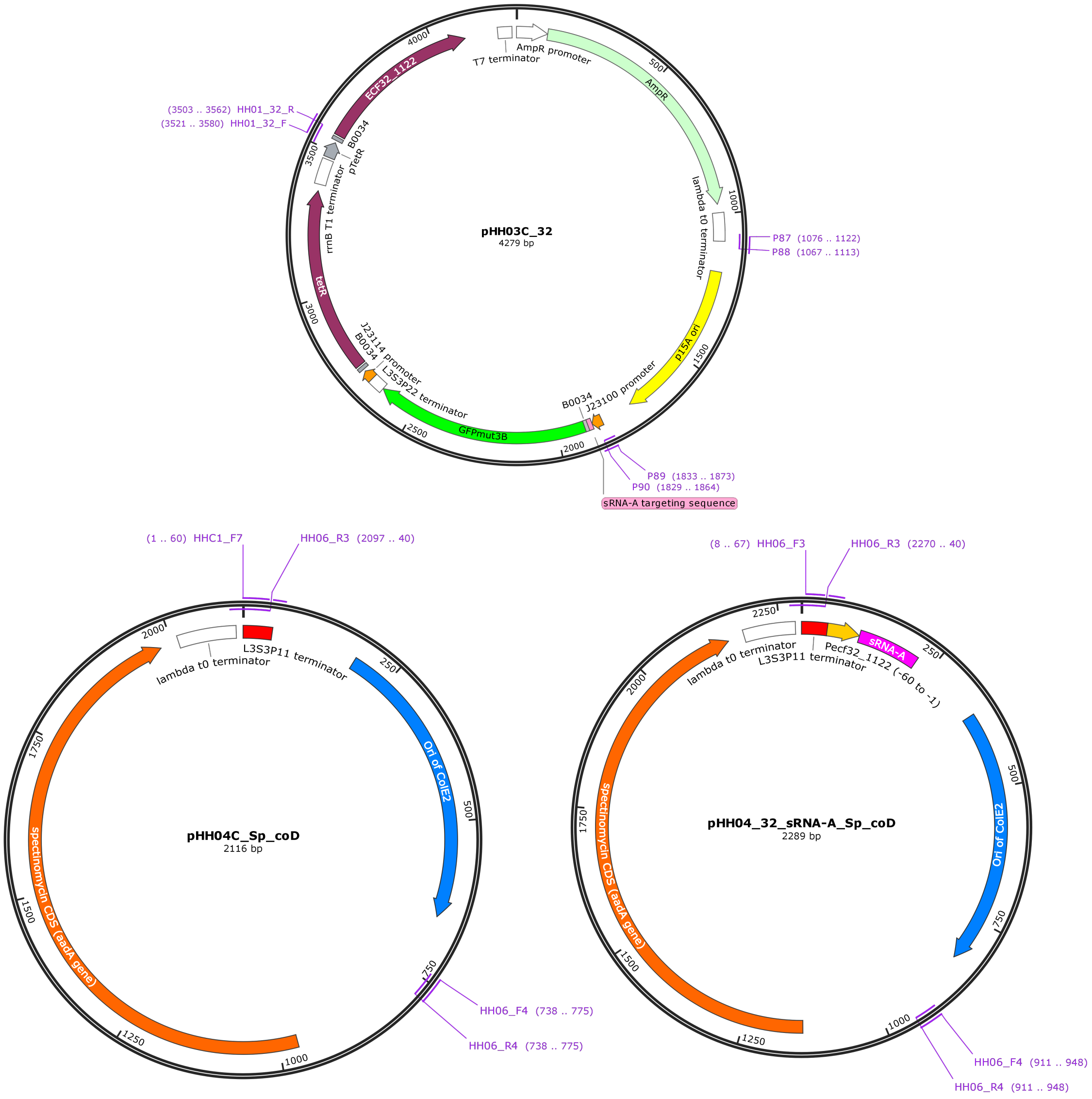

Supplementary Figure 2: The maps of pHH03 and pHH04 plasmids. The assembled fragments can be found in Supplementary Table 1. The maps are prepared by the SnapGene software.

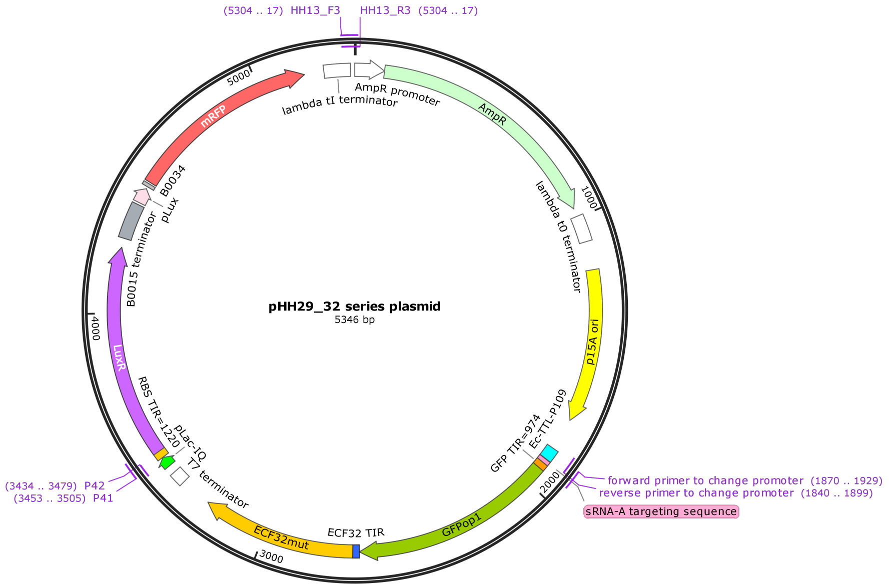

Supplementary Figure 3: The map of pHH29 series plasmid. The assembled fragments can be found in Supplementary Table 1. The operon's promoter and the RBS of *ECF32mut* gene were changed according to Supplementary Table 3. The map is prepared by the SnapGene software.

Construct	Plasmid 1	Plasmid 2	Circuit	Figure
pHH3414	pHH03C_32	pHH04_32_sRNA-A_Sp_coD	w/ sRNA	4A
pHH3413	pHH03C_32	pHH04C_Sp_coD	w/o sRNA	4B

Supplementary Table 2: The genetic circuit used in Figure 4 was built by co-transforming the two indicated plasmids into *E. coli* DIAL strain JTK-160J [1].

Construct	Plasmid 1	Plasmid 2	TX^a	ECF32 TIR^b	operon promoter
pHH290479	pHH29_32_HG18	pHH04_32_sRNA-A_Sp_coD	reg	565	J23116
pHH290480	pHH29_32_HG19	pHH04_32_sRNA-A_Sp_coD	reg	1127	J23116
pHH2904110	pHH29_32_HG02	pHH04_32_sRNA-A_Sp_coD	reg	6474	J23116
pHH2904106	pHH29_32_HG35	pHH04_32_sRNA-A_Sp_coD	reg	565	Ec-TTL-P109
pHH2904107	pHH29_32_HG36	pHH04_32_sRNA-A_Sp_coD	reg	1127	Ec-TTL-P109
pHH290498	pHH29_32_HG27	pHH04_32_sRNA-A_Sp_coD	reg	6474	Ec-TTL-P109
pHH290461	pHH29_32_HG18	pHH04C_Sp_coD	unreg	565	J23116
pHH290462	pHH29_32_HG19	pHH04C_Sp_coD	unreg	1127	J23116
pHH290443	pHH29_32_HG02	pHH04C_Sp_coD	unreg	6474	J23116
pHH2904112	pHH29_32_HG35	pHH04C_Sp_coD	unreg	565	Ec-TTL-P109
pHH2904113	pHH29_32_HG36	pHH04C_Sp_coD	unreg	1127	Ec-TTL-P109
pHH290488	pHH29_32_HG27	pHH04C_Sp_coD	unreg	6474	Ec-TTL-P109

Supplementary Table 3: The genetic circuit used in Figure 5 was built by co-transforming the two indicated plasmids into *E. coli* DIAL strain JTK-160J [1]. ^{a}, A regulated or unregulated TX device was noted as reg or unreg, respectively. ^{b}, The translation initiation rate (TIR) was predicted by the RBS calculator [2]. The TIR of the *GFPop1* gene's RBS is 974.

Construct	Plasmid 1	Plasmid 2	Supplementary Figure
pHH1215A	pHH01_15	pHH02_15UP436	4 , 5A , 5D
pHH1222A	pHH01_22	pHH02_22UP1147	4 , 5B , 5E
pHH1232A	pHH01_32	pHH02_32_1122	4 , 5C , 5F

Supplementary Table 4: A genetic circuit for functional test of extracytoplasmic function (ECF) sigma factors and their cognate promoters was built by co-transforming the two indicated plasmids into *E. coli* DIAL strain JTK-160J [1]. The plasmid diagram can be found in Supplementary Figure 4 and the results of functional test are in Supplementary Figure 5A-F.

Supplementary Note 1

Selection of ECF for controller implementation

A set of genetic circuits were constructed to test the function of candidate ECF sigma factors and their cognate promoters (Supplementary Figure 4). The circuits were built on two separate plasmids (see Supplementary Figure 1 and Supplementary Table 4):

- a p15A plasmid with ampicillin resistance cassette to express ECF protein, and
- a ColE2 plasmid with chloramphenicol resistance cassette to express sfGFP (superfolder GFP) protein when ECF protein is present.

TetR-regulated *ECF* gene can be induced by TetR's effector anhydrotetracycline (aTc). These circuits are only different in the *ECF* gene and the pECF promoter. Specifically, the candidate *ECF* genes are *ecf15_436*, *ecf22_4450*, and *ecf32_1122*. The candidate pECF promoters are pECF15_up436, pECF22_up1147, and pECF32_1122, respectively. The *ECF* genes and their cognate pECF promoters must be used in pair. The DNA fragment encoding the respective *ECF* gene and the T7 terminator was cloned from the pVRa plasmid [3] bearing the respective *ECF* gene. The *sfGFP* gene cassette, including the respective pECF promoter, the ribosome binding site (RBS), the *sfGFP* gene, and the *rrnB* T1 terminator, was cloned from the pVRb plasmid [3] bearing the respective pECF promoter. The RBS of the *sfGFP* gene cassette is a strong synthetic RBS originally designed in the pVRb plasmid. The pVRa and pVRb plasmids were purchased from Addgene. The two plasmids of each circuit were co-transformed into *E. coli* DIAL JTK-160J [1] to allow ColE2-plasmid to self-replicate.

Functional test of ECF sigma factors and their cognate promoters was conducted by adding aTc to induce ECF sigma factor to observe how sfGFP expresses. The temporal response of sfGFP expression and cell growth data are shown in Supplementary Figure 5. Among the three candidate ECF sigma factors, only ECF32 sigma factor did not impact cell growth significantly (Supplementary Figure 5C). In addition, in accordance with our design requirement for a large dynamic range, sfGFP expression driven by pECF32 promoter did not saturate even when 1000 nM aTc was added (Supplementary Figure 5F). Therefore, we selected ECF32 sigma factor and its cognate promoter pECF32 as the key biological parts to build the sRNA-mediated quasi-integral controller. Note that the DNA sequences of ECF sigma factors and their cognate promoters used in the functional test were readily available from the pVRa and pVRb plasmids without modification. When using ECF32 sigma factor and pECF32 promoter to construct the regulated TX devices, a proper DNA sequence modification is needed and indicated in Supplementary Note 2.

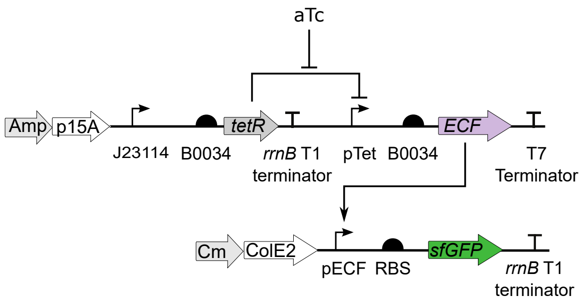

Supplementary Figure 4: A genetic circuit for functional test of ECF sigma factors and their cognate promoters. The genetic *locus* labeled as *ECF* gene is cloned in either the *ecf15_436*, *ecf22_4450*, or *ecf32_1122* gene. The genetic *locus* labeled as pECF promoter is cloned in either the pECF15_up436, pECF22_up1147, or pECF32_1122 promoter. The *ECF* gene must be cloned in pair with its cognate pECF promoter. ECF protein expression is regulated by TetR repressor and its effector aTc. The respective ECF proteins bind to their cognate promoters to express the *sfGFP* gene.

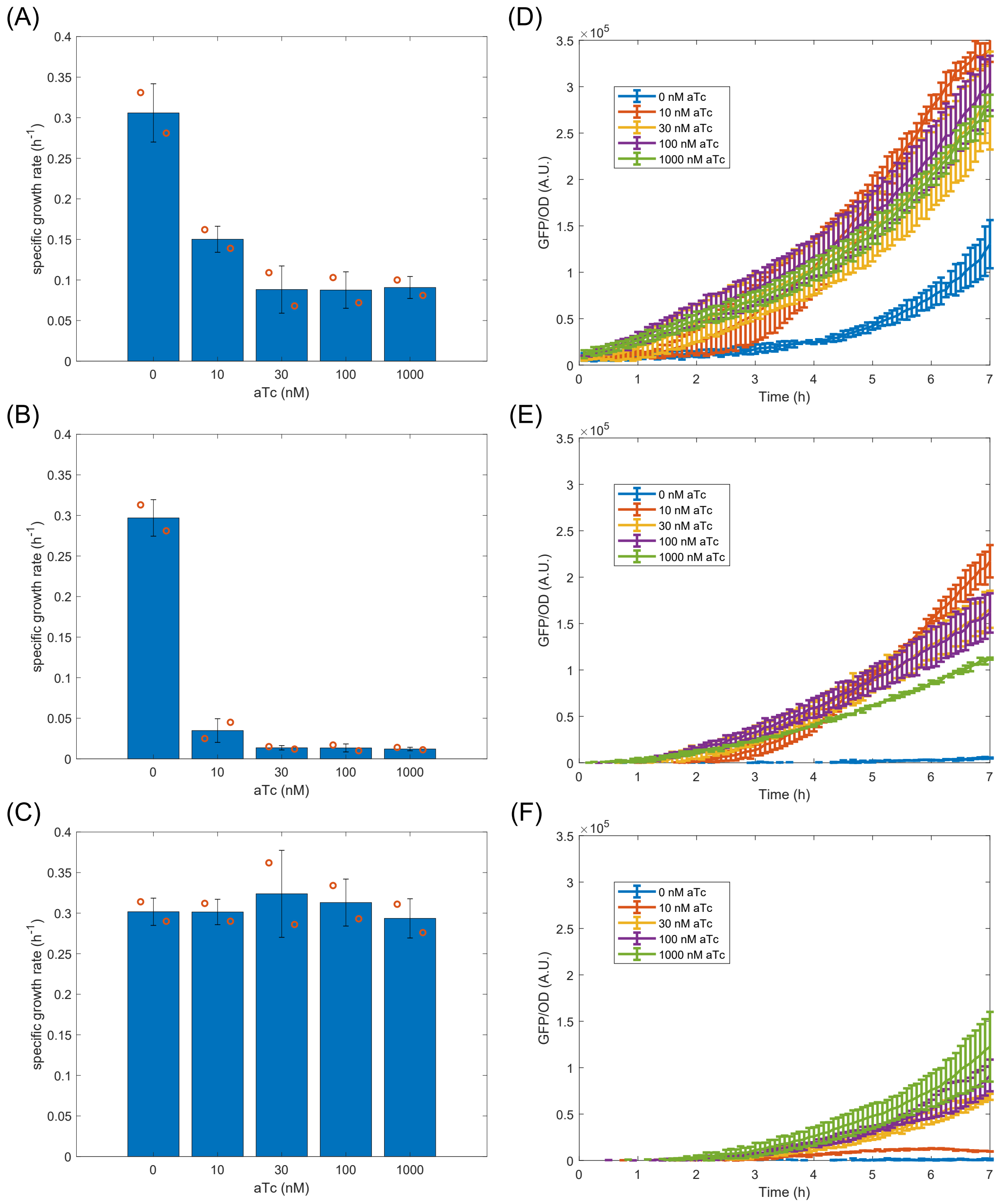

Supplementary Figure 5: Functional test of three ECF sigma factors and their cognate promoters. (A-C) Specific growth rates of cells expressing the *sfGFP* gene with the ECF15_436, ECF22_4450, and ECF32_1122 sigma factors and their respective cognate promoters. Specific value of an independent experiment presents as an orange circle. (D-F) The temporal response of *sfGFP* expression by the ECF15_436, ECF22_4450, and ECF32_1122 sigma factors and their cognate promoters at the indicated aTc inductions. Data with error bars represent mean values with standard deviations from two independent experiments by microplate photometer. Source data are provided as a Source Data file.

Supplementary Note 2

DNA sequences

To transcribe sRNA-A from pECF32 promoter's transcription start site (TSS), DNA sequence of pECF32 promoter used in the post-TX controller ranges from -60 to -1 (Supplementary Figure 2), instead from -60 to +20 as used in the functional test (Supplementary Figure 1). The *gfpmut3B* and *ecf32* genes used in a TX device were re-designed with the operon calculator [4] in order to remove unwanted intrinsic terminators and ribosome pausing sites while keeping their amino acid sequences unchanged with a balanced synonymous codon usage table. The two genes are re-named as *GFPop1* and *ECF32mut*, respectively, as shown in Supplementary Figure 3. The following DNA sequences are in FASTA format and are arranged in an order from the 5'-end to the 3'-end in the respective DNA sections of the plasmid maps (Supplementary Figure 2-3).

pECF32 promoter and sRNA-A in pHH04_32_sRNA-A_Sp_coD

>pECF32 promoter (-60 to -1)
gcgactttatttaacagcggcatggccagggaaccgatgcgtcaatcgca
ccacacaatg

>sRNA-A (sRNA-A tag in bolded font)
tttgacctgatgagtccgtgaggacgaaacgagctagctcgtc**gtcaaag**
tttgttatatttgtagaaatattttattcgcccccggaagatcattccgg
gggctttttttatt

A bi-cistronic operon

The following DNA sequences are used for the respective biological parts of the bi-cistronic operon in the pHH29 series plasmids (Supplementary Figure 3). The operon's promoter and the ribosome binding sites of *ECF32mut* gene were changed according to Supplementary Table 3.

>operon's promoter J23100
ttgacggctagctcagtcctaggtacagtgctagc

>operon's promoter J23116
ttgacagctagctcagtcctagggactatgctagc

>operon's promoter Ec-TTL-P109 (underlined)
ggcgcgccaaaaagagtattgacttcaggaaatttttctgcataattatttca

>sRNA-A targeting sequence
ataacaaactttgac

>RBS TIR=974 of *GFPop1* gene
acttgggtcttataggatgatta

>*GFPop1* gene
atgcgtaaaggagaagaactgttcactggagttgtcccaattcttgttga
attagatggtgatgttaatgggcacaaattttctgtgagtggagagggtg
aaggtgatgcgacatacggcaaactgacccttaaatttatttgtactacc
ggcaagctacctgttccttggccgacacttgtcactactttcggttatgg

tgttcaatgttttgcgagatacccagatcatatgaaacagcatgactttt
tcaaaagtgccatgcccgaaggttatgtacaggaaagaactatatttttc
aaagatgacgggaactacaaaacacgtgctgaagttaaatttgagggtga
tactctggtaaatagaatcgaattaaaaggtattgattttaaagaagatg
gcaacattcttggtcacaagctggaatacaactacaactcacacaatgtt
tacatcatggcagacaaacaaaaaaatggcatcaaagttaacttcaaaat
tcgacacaacattgaagatggaagcgttcaactggcagaccattatcaac
agaatactccgattggcgatggccctgtactcttaccagacaaccattac
ctgtccacacaatctgccctttcgaaagatccgaacgaaaagagagatca
catggtcctgcttgagtttgtaacagctgctgggattacacatggtatgg
atgagctatacaaataataa

>RBS TIR=471 of *ECF32mut* gene
AGGAAATTTGATTTCTTAAGTTG

>RBS TIR=565 of *ECF32mut* gene
AGGAAATTTGATTTCTTGAGATA

>RBS TIR=1127 of *ECF32mut* gene
GTCGCTACTATAAGAGCATAAGTCTC

>RBS TIR=6474 of *ECF32mut* gene
CAACCTTTCACGGAGTAGGACATCAA

>*ECF32mut* gene
atgaccgagattcatctgcagaccacagaaagcaccagcgttaatgatgg
tctgccgctgaatattgattgggaaggtatttttcgtgagcatggccgtc
gtgtccatcattttattcgtaaacgtgtcagccatcgagaagatgcagaa
gatctggaacagatgacctggctggaagttcttcgtaatagggataaatt
tgccggcgcatcacgtccggaaacctgggtttttggtatagcactgaacc
tggtgcgtaatcatttccgtctgcagagcggtcgtccgcgttgtgatgaa
ctggaagatgatattattctgacccagggtgatgatccgagccatattac
cgaatatcagcgtctgctgaatagcaccctgaatagtattgcaaacctgc
cggaagatacccgtcgcctgctgaacatgctggttgaaaaagatggtagc
tatcaggcaattgcagcccatctgaatattccaattggcaccgttcgtag
ccgtctgagccgtgcacgtgttaccctgaaacagagcgtttttagctaa

>3'-end of the operon (T7 terminator in bolded font)
ggatccaagcttgcggccgcactcgagcaccaccaccaccaccactgaga
tccggctgctaacaaagcccgaaaggaagctgagttggctgctgccaccg
ctgagcaataa**ctagcataaccccttggggcctctaaacgggtcttgagg**
ggttttttg

Resource Competitor

The resource competitor was cloned from the one used in our previous work [5] but replaced the RBS of *luxR* gene from a strong BBa_B0034 to a weaker RBS with TIR 1220.

> pLac-IQ promoter (underlined)
tggtgcaaaacctttcgcggtatggcatgatagcgcct

> RBS (TIR=1220)
AATACAATGCTCTAGGTAGAAGCAA

> *luxR* gene
atgaaaaacataaatgccgacgacacatacagaataattaataaaattaa
agcttgtagaagcaataatgatattaatcaatgcttatctgatatgacta
aaatggtacattgtgaatattatttactcgcgatcatttatcctcattct
atggttaaatctgatatttcaatcctagataattaccctaaaaaatggag
gcaatattatgatgacgctaatttaataaaatatgatcctatagtagatt
attctaactccaatcattcaccaattaattggaatatatttgaaaacaat
gctgtaaataaaaaatctccaaatgtaattaaagaagcgaaaacatcagg
tcttatcactgggtttagtttccctattcatacggctaacaatggcttcg
gaatgcttagttttgcacattcagaaaaagacaactatatagatagttta
tttttacatgcgtgtatgaacataccattaattgttccttctctagttga
taattatcgaaaaataaatatagcaaataataaatcaaacaacgatttaa
ccaaaagagaaaaagaatgtttagcgtgggcatgcgaaggaaaaagctct
tgggatatttcaaaaatattaggttgcagtgagcgtactgtcactttcca
tttaaccaatgcgcaaatgaaactcaatacaacaaaccgctgccaaagta
tttctaaagcaattttaacaggagcaattgattgcccatactttaaaaat
taataa

> flanking sequence of BBa_B0015 terminator (bolded font)
cactgatagtgctagtgtagatcactactagag**ccaggcatcaaataaaa**
cgaaaggctcagtcgaaagactgggcctttcgttttatctgttgtttgtc
ggtgaacgctctctactagagtcacactggctcaccttcgggtgggcctt
tctgcgtttatatactagag

> pLux promoter (TSS uppercased and underlined)
acctgtaggatcgtacaggtttacgcaagaaaatggtttgttatagtcga
at<u>A</u>aa

> flanking sequence of BBa_B0034 RBS (bolded font)
tactagag**aaagaggagaaa**tactag

> *mRFP1* gene
atggcttcctccgaagacgttatcaaagagttcatgcgtttcaaagttcg
tatggaaggttccgttaacggtcacgagttcgaaatcgaaggtgaaggtg
aaggtcgtccgtacgaaggtacccagaccgctaaactgaaagttaccaaa
ggtggtccgctgccgttcgcttgggacatcctgtccccgcagttccagta
cggttccaaagcttacgttaaacacccggctgacatcccggactacctga
aactgtccttcccggaaggtttcaaatgggaacgtgttatgaacttcgaa
gacggtggtgttgttaccgttacccaggactcctccctgcaagacggtga
gttcatctacaaagttaaactgcgtggtaccaacttcccgtccgacggtc
cggttatgcagaaaaaaaccatgggttgggaagcttccaccgaacgtatg
tacccggaagacggtgctctgaaaggtgaaatcaaaatgcgtctgaaact
gaaagacggtggtcactacgacgctgaagttaaaaccacctacatggcta
aaaaaccggttcagctgccgggtgcttacaaaaccgacatcaaactggac
atcacctcccacaacgaagactacaccatcgttgaacagtacgaacgtgc
tgaaggtcgtcactccaccggtgcttaataa

> flanking sequence of lambda tI terminator (bolded font)
cgctgatagtgctagtgtagatcgctactagagtactagctctagatgcg
gccgcgaattccagaaatca**tccttagcgaaagctaaggatttttttat**

ctgaaatgcctctgtaacagagcattagcgcaaggtgatttttgtcttct
tgcgctaattttt

Supplementary Note 3

RT-qPCR primers

> RTp4 primer for cDNA synthesis of the *mRFP1* transcript
`agcaccggtggagtgacgac`

Gene	forward primer	reverse primer	PCR efficiency[a]
cysG[b]	ttgtcggcggtggtgatgtc	atgcggtgaactgtggaataaacg	1.03±0.06
mRFP1	tggcttcctccgaagacg	cggtctgggtaccttcgtac	0.96±0.05

Supplementary Table 5: The qPCR primer pairs for the reference *cysG* gene and the target *mRFP1* gene. [a], The PCR efficiency [6] was determined experimentally with three biological replicates, each with three technical replicates. [b], The qPCR primer pair is the same as the one in [7].

.

Supplementary Note 4

Temporal response data from microplate photometer

The fluorescence and OD data of temporal response obtained with microplate photometer are provided in this section. These data were used to obtain dose responses in Figure 4-6 in the main text. Unless otherwise specified, the growth condition and measurement protocol used here are identical to the ones described in the methods section of the main text. The number of biological and technical replicates can be found in the captions of each figure.

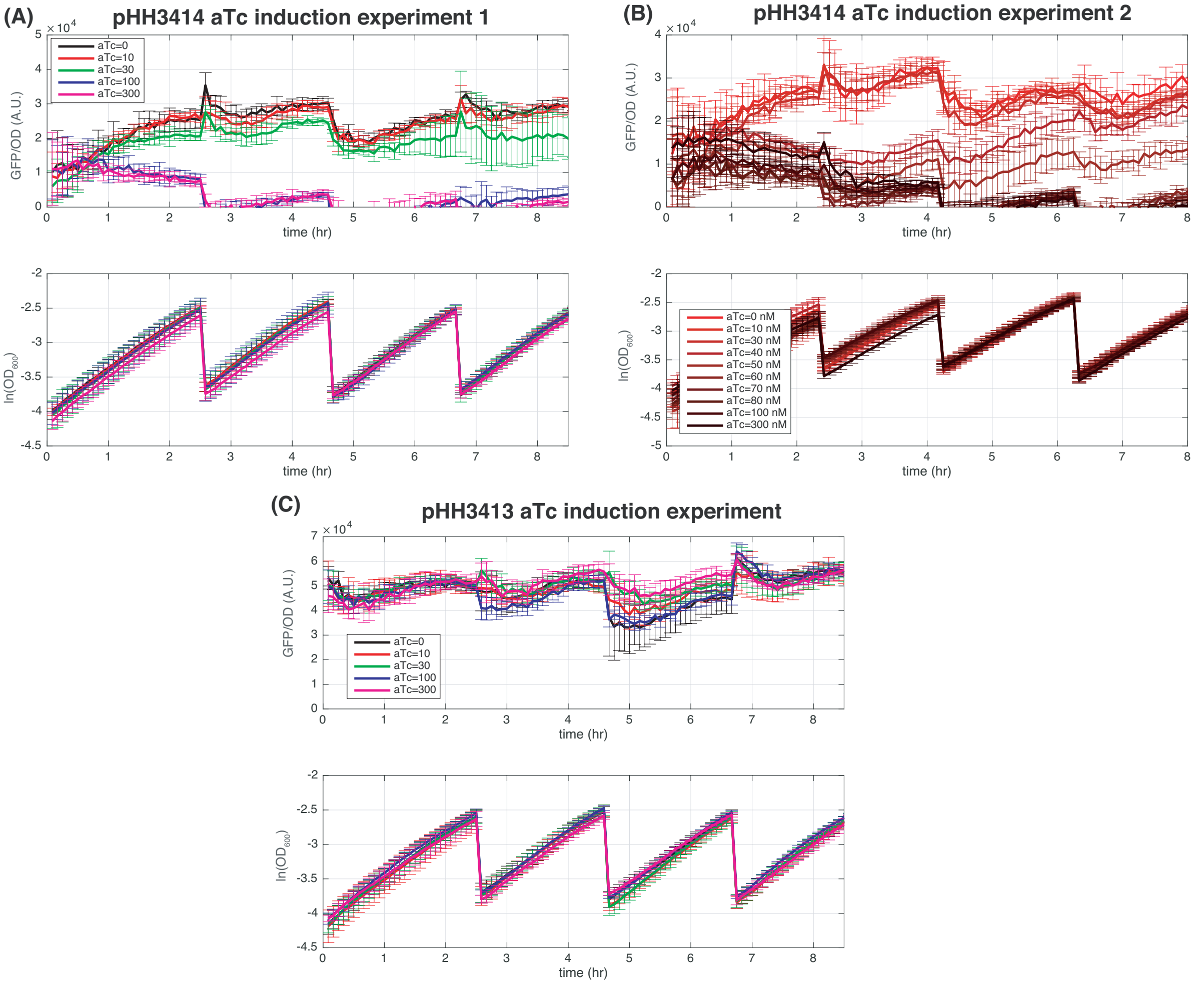

Supplementary Figure 6: Temporal response of GFP and OD_{600} used to obtain the dose response curves of the sRNA silencing experiments in Figure 4 of the main text. Figure 4a was obtained by combining experimental data from the two independent experiments shown in panels (A) and (B) here. The temporal response in panel (C) was used to derive the dose response curve in Figure 4b. Error bars indicate standard deviations from two biological replicates, each with two technical replicates. Source data are provided as a Source Data file.

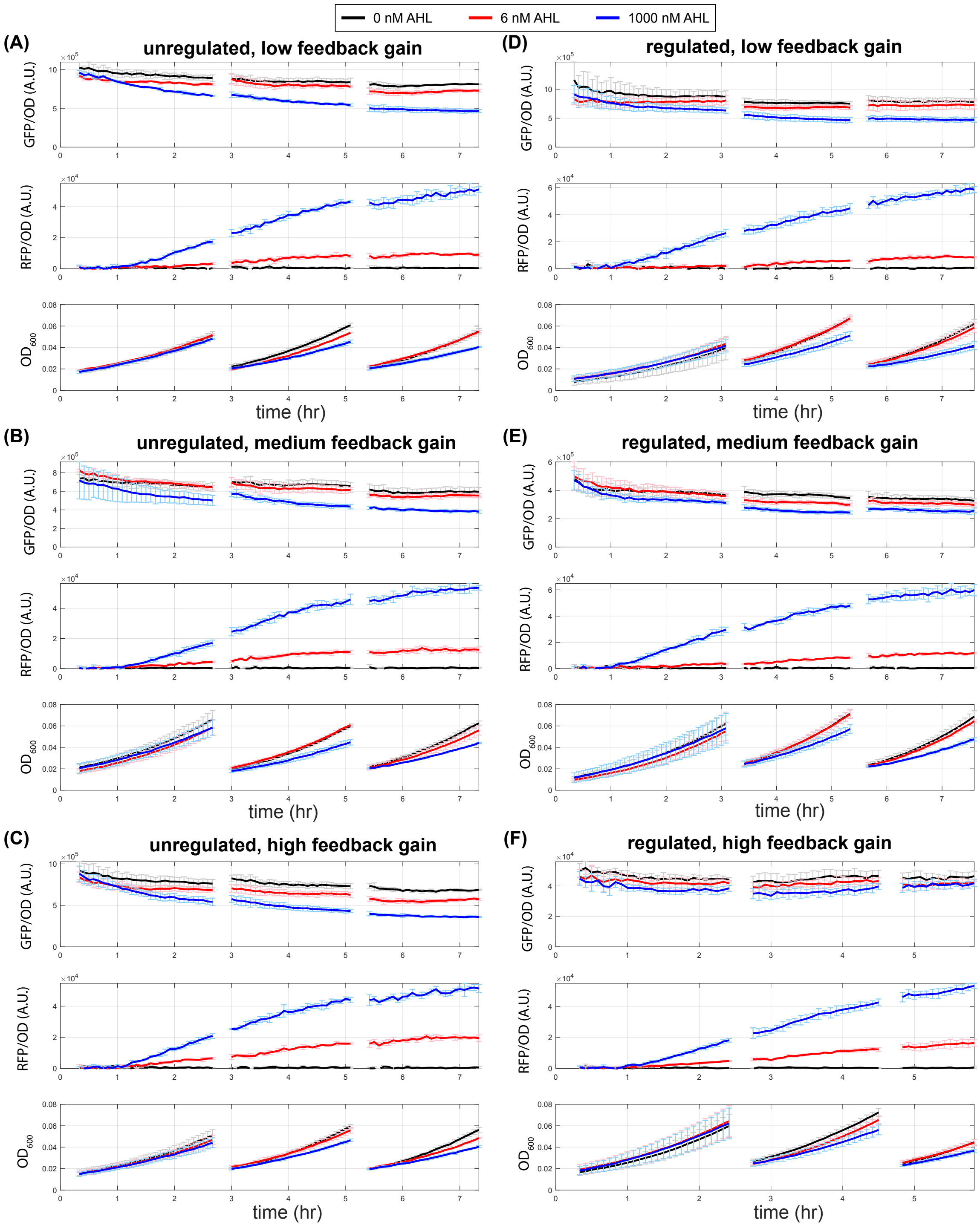

Supplementary Figure 7: Temporal response of GFP, RFP and OD_{600} used to obtain nominal output and robustness of regulated and unregulated devices driven by the Ec-TTL-P109 promoter in Figure 5 of the main text. Error bars are standard deviations from three biological replicates with a single technical replicate. The only exception is the high-gain regulated device, whose error bars are standard deviations from six replicates, including three biological replicates, each with two technical replicates. (A)-(C) The unregulated TX devices with low, medium, and high gain, respectively. (D)-(E) The regulated TX devices with low, medium, and high gain, respectively. The respective AHL concentrations are indicated in the legend at the top. Source data are provided as a Source Data file.

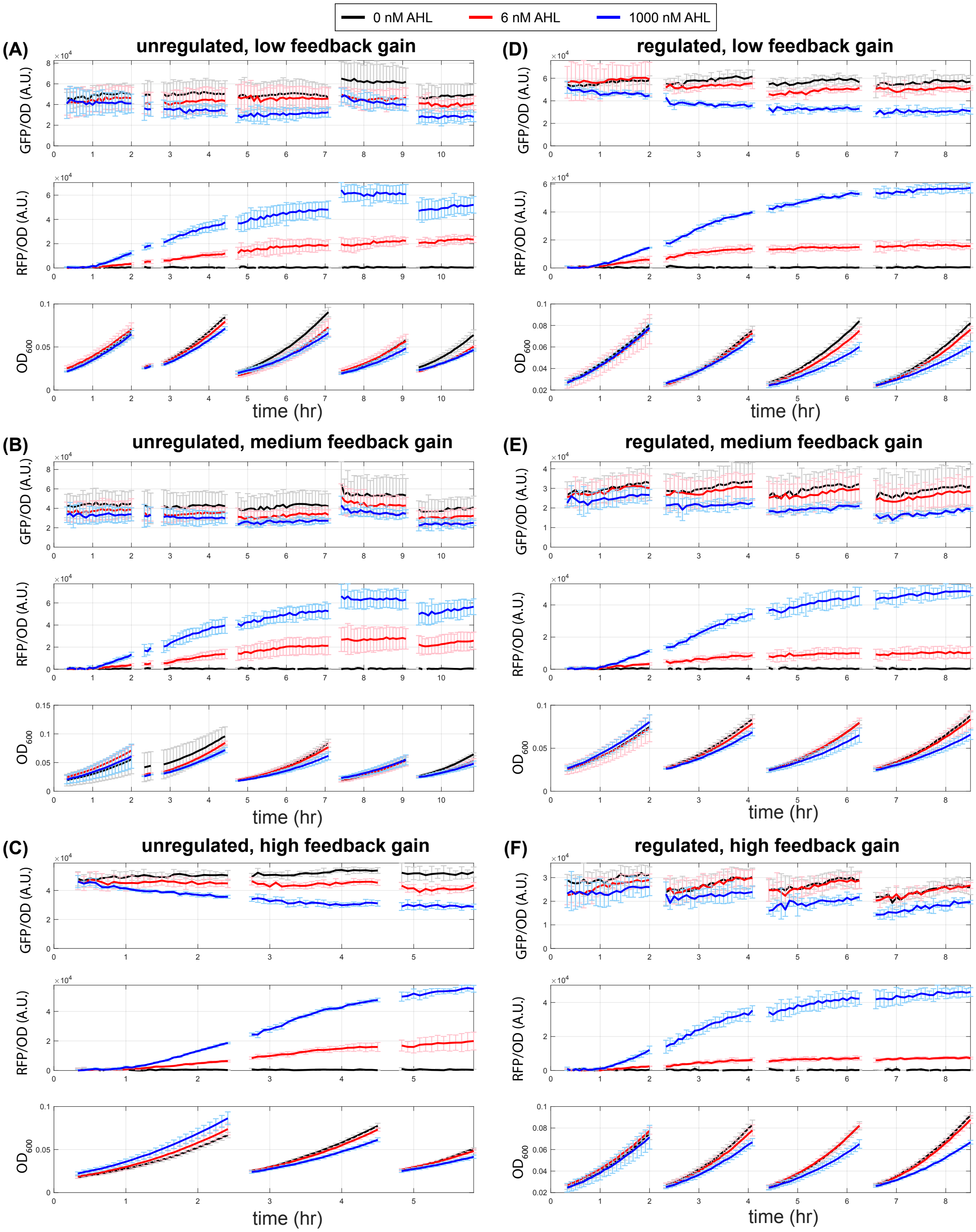

Supplementary Figure 8: Temporal response of GFP, RFP and OD_{600} used to obtain nominal output and robustness of regulated and unregulated devices driven by the BBa_J23116 promoter in Figure 5 of the main text. Error bars represent standard deviations from three biological replicates with a single technical replicate. The only exception is the high-gain unregulated device, whose error bars are standard deviations for six replicates, including three biological replicates, each with two technical replicates. (A)-(C) The unregulated TX devices with low, medium, and high gain, respectively. (D)-(E) The regulated TX devices with low, medium, and high gain, respectively. The respective AHL concentrations are indicated in the legend at the top. Source data are provided as a Source Data file.

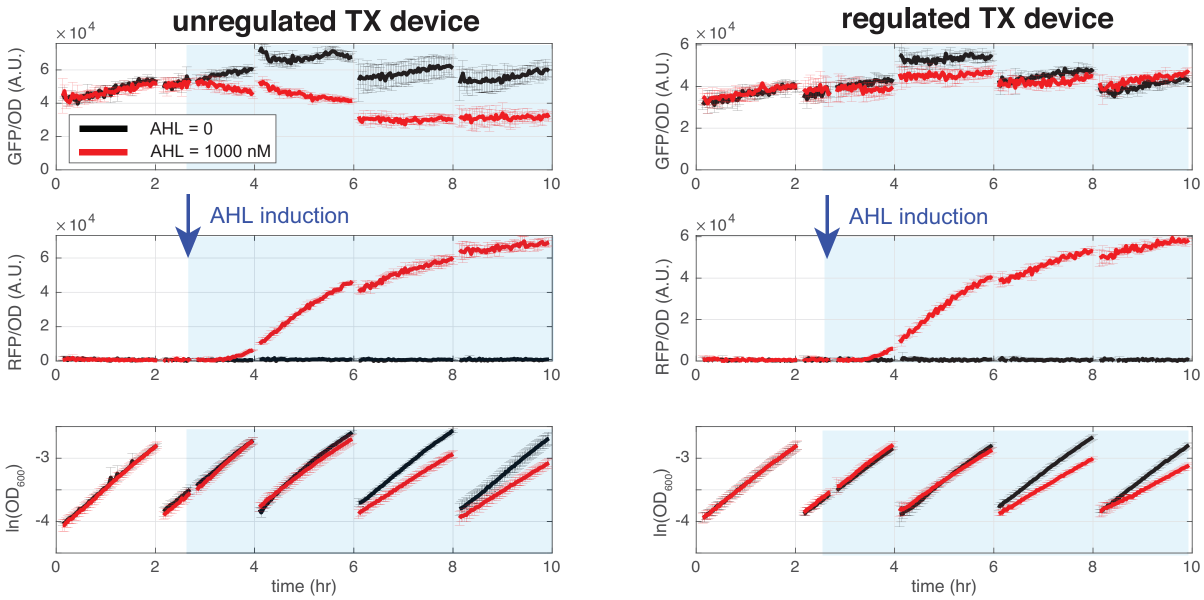

Supplementary Figure 9: Temporal response data in Figure 6 of the main text without normalizing GFP levels. Error bars are standard deviations from three biological replicates. Source data are provided as a Source Data file.

Supplementary Note 5

Steady state data from microplate photometer

Temporal data obtained from microplate photometer presented in Supplementary Figures 7-8 were used to obtain the steady state responses of the TX devices. The last GFP per OD, RFP per OD, and OD values in Supplementary Figures 7-8 were used as steady state values. They were at least 6 hours after AHL induction. The OD_{600} data of the last batch were used to fit an exponential function to decide specific growth rate.

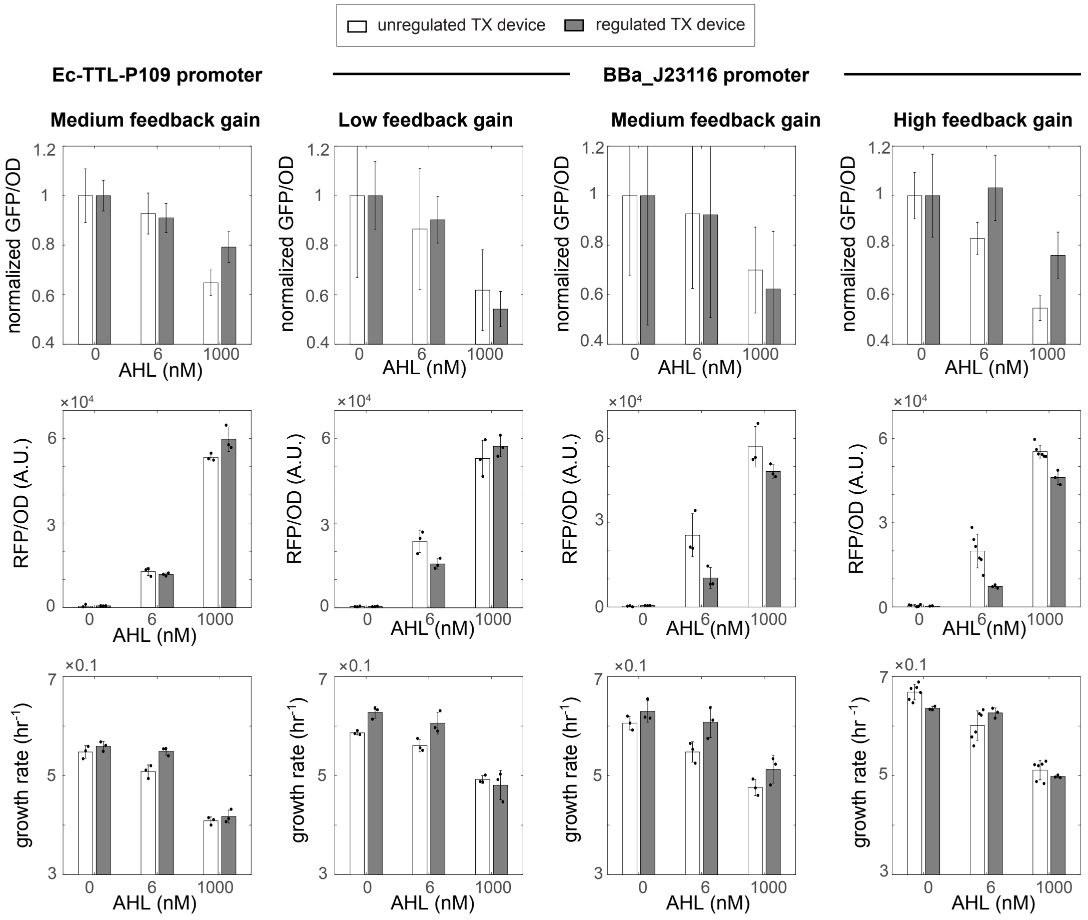

Supplementary Figure 10: Steady-state expression levels of GFP and RFP and growth rates for the regulated and unregulated TX devices. The devices are driven by Ec-TTL-P109 and BBa_J23116 promoter, respectively, with the indicated feedback gain. Error bars are standard deviations. The unregulated device using BBa_J23116 promoter and high feedback gain has six replicates including three biological replicates, each with two technical replicates. Other devices have three biological replicates with a single technical replicate. Temporal data used to obtain steady state response here are in Supplementary Figures 7-8. Specific value of an independent experiment presents as a black dot. Source data are provided as a Source Data file.

Supplementary Note 6

Steady state data from flow cytometry

Flow cytometry data were collected at 6 hours after AHL induction. The singlet gating strategy is shown in Supplementary Figure 11. The fluorescence scatter plots, histograms and their important statistics are provided in Supplementary Figures 12-13. The correlation coefficient between RFP and GFP are listed in Supplementary Tables 6-7. For both the regulated and the unregulated devices, GFP and RFP were weakly positively correlated. At least the following two aspects of cell-cell variability may contribute to this weak positive correlation between GFP and RFP.

- GFP and RFP are on the same plasmid. Therefore, a stochastic increase (decrease) in plasmid copy number leads to simultaneous increases (decreases) in both GFP and RFP.
- Similarly, GFP and RFP use the same pool of cellular resources. As a consequence, a stochastic increase (decrease) in the total number of ribosomes leads to increases (decreases) in both GFP and RFP expression.

AHL (nM)	unregulated # 1	unregulated # 2	unregulated # 3
0	0.52	0.54	0.48
1000	0.33	0.41	0.41

Supplementary Table 6: Correlation between RFP and GFP for the unregulated TX device

AHL (nM)	regulated # 1	regulated # 2	regulated # 3
0	0.16	0.19	0.21
1000	0.30	0.41	0.42

Supplementary Table 7: Correlation between RFP and GFP for the regulated TX device

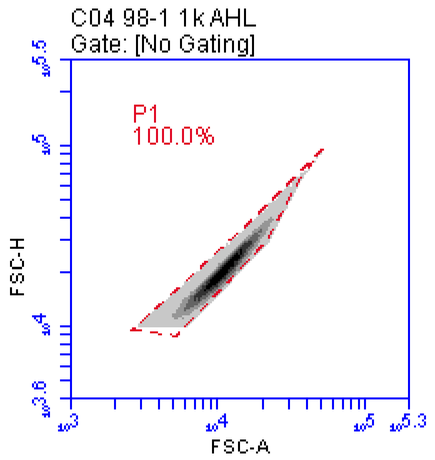

Supplementary Figure 11: The gating strategy of flow cytometry. Singlet events were gated as the P1 population in a FSC-H vs. FSC-A plot and the events outside P1 were not collected. At least 120,000 singlet events were collected.

Sample Name	Count	Mean : FL1-H	Median : FL1-H	Geometric Mean : FL1-H	Robust CV : FL1-H	Median : FL4-H
A01 43-1 0 AHL.fcs	296927	21300	17344	16007	65.1	174
C01 43-1 1k AHL.fcs	162473	10380	8706	7983	75.9	18557

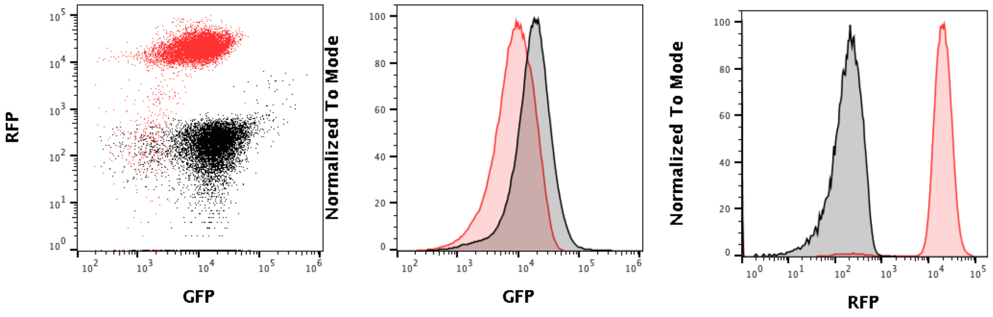

Sample Name	Count	Mean : FL1-H	Median : FL1-H	Geometric Mean : FL1-H	Robust CV : FL1-H	Median : FL4-H
A02 43-2 0 AHL.fcs	249429	21086	16995	15389	66.9	177
C02 43-2 1k AHL.fcs	177237	11385	9811	8551	76.7	17891

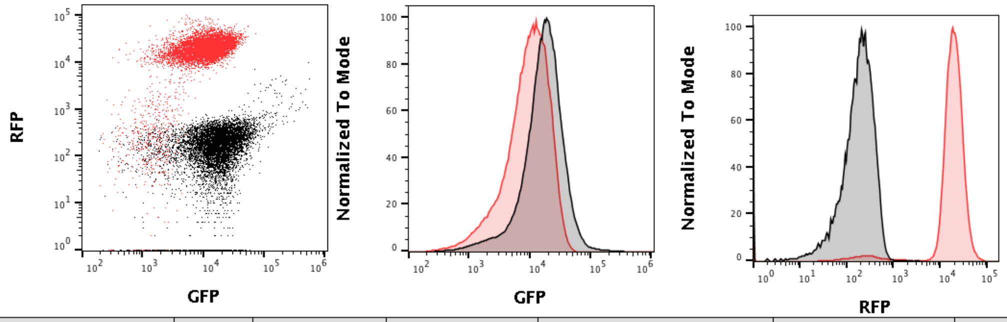

Sample Name	Count	Mean : FL1-H	Median : FL1-H	Geometric Mean : FL1-H	Robust CV : FL1-H	Median : FL4-H
A03 43-3 0 AHL.fcs	252526	23151	18672	17439	64.4	169
C03 43-3 1k AHL.fcs	163886	13604	11843	10572	72.2	18258

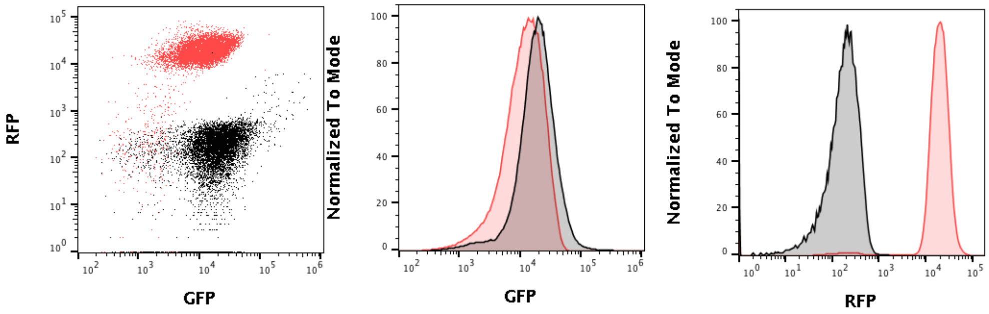

Supplementary Figure 12: Flow cytometry data of the unregulated device at steady state. GFP and RFP scatter plots (the left column), GFP histograms (the middle column) and RFP histograms (the right column) for three biological replicates are presented. Important statistics, including cell count, GFP (FL1-H) mean, median and geometric mean, GFP (FL1-H) robust CV, and RFP (FL4-H) median for each sample are tabulated at the top of the figures for each sample.

	Sample Name	Count	Mean : FL1-H	Median : FL1-H	Geometric Mean : FL1-H	Robust CV : FL1-H	Median : FL4-H
▯	A04 98-1 0 AHL.fcs	246676	14212	11400	10441	82.4	179
▯	C04 98-1 1k AHL.fcs	166597	17145	13761	13118	79.7	15903

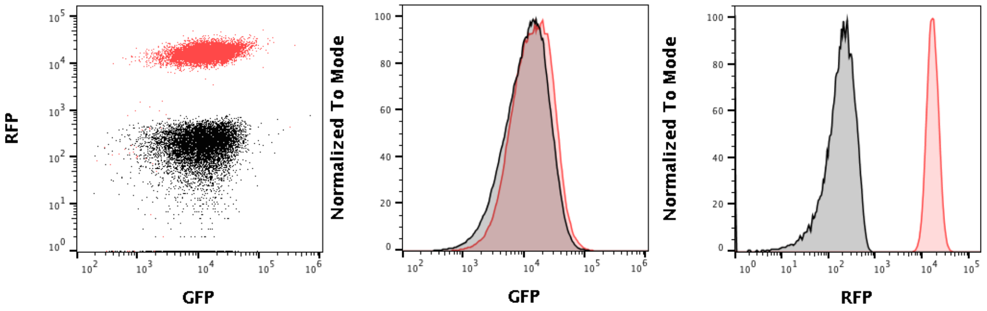

	Sample Name	Count	Mean : FL1-H	Median : FL1-H	Geometric Mean : FL1-H	Robust CV : FL1-H	Median : FL4-H
▯	A05 98-2 0 AHL.fcs	262739	17904	15469	14140	67.8	185
▯	C05 98-2 1k AHL.fcs	167103	16167	13012	12058	81.1	16362

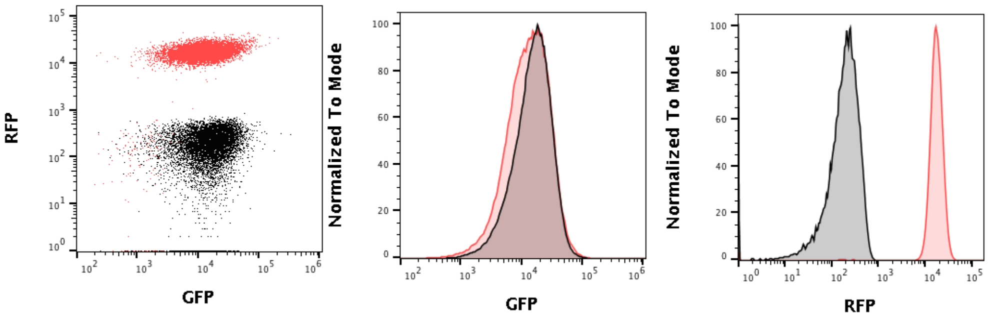

	Sample Name	Count	Mean : FL1-H	Median : FL1-H	Geometric Mean : FL1-H	Robust CV : FL1-H	Median : FL4-H
▯	A06 98-3 0 AHL.fcs	221547	17348	15004	13419	70.7	176
▯	C06 98-3 1k AHL.fcs	128651	18196	16029	13847	67.7	16562

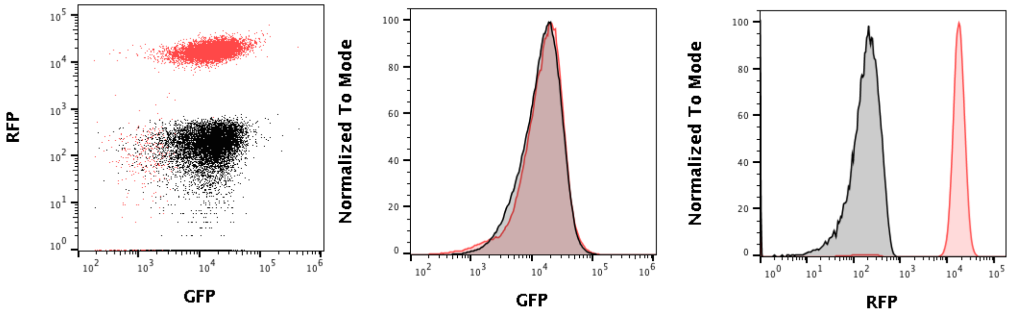

Supplementary Figure 13: Flow cytometry data of the regulated device at steady state. GFP and RFP scatter plots (the left column), GFP histograms (the middle column) and RFP histograms (the right column) for three biological replicates are presented. Important statistics, including cell count, GFP (FL1-H) mean, median and geometric mean, GFP (FL1-H) robust CV, and RFP (FL4-H) median for each sample are tabulated at the top of the figures for each replicate.

Supplementary Note 7

Derivation of the sRNA-mediated feedback model

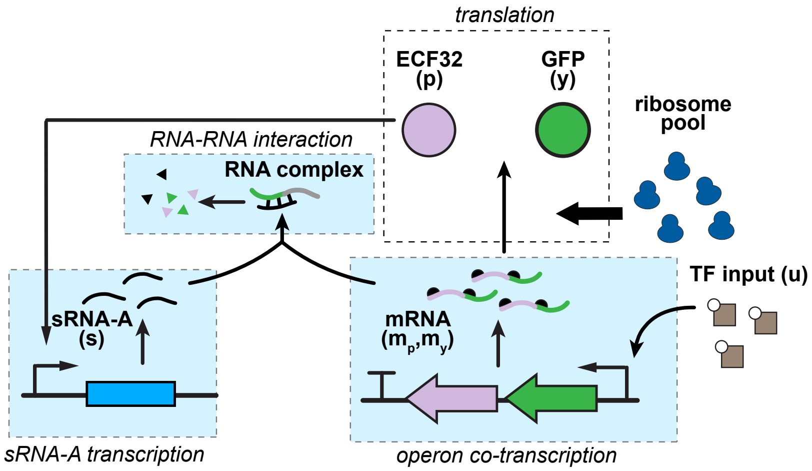

Supplementary Figure 14: Circuit diagram of a TX device regulated by sRNA-mediated feedback.

Here we derive a simple mathematical model to demonstrate the working principle of the sRNA-mediate feedback circuit. The circuit is depicted in Supplementary Figure 14, where m, s, p and y each stands for the species of GFP/ECF32 mRNA co-transcript, sRNA-A, ECF32 protein and GFP protein. We use italic x to represent the concentration of species x (roman). For generality, we consider a transcription factor (TF) u regulating the co-transcription of GFP/ECF32 genes. Their transcription rate is related to the concentration of TF input u by a function $TDH(u)$, where T is the transcription rate constant per DNA copy, D is the DNA plasmid copy number, and $H(\cdot) : \mathbb{R}_{\geq 0} \mapsto [0, 1]$ describes the extent to which GFP/ECF32 gene transcription is activated/repressed. In a situation where the GFP/ECF promoter is constitutive, we set $H(u) \equiv 1$. We assume that the amount of transcriptional machinery, chiefly RNA polymerase, is abundant [5, 8–10] so that transcription rate of $\mathrm{m_p}/\mathrm{m_y}$ is determined by promoter strength, plasmid copy number and its own TF input u and is therefore completely decoupled from that of the resource competitor. The mRNA co-transcripts are decayed (i.e., degraded and diluted) at a rate constant δ_m. These processes can be described by the reactions

$$\mathrm{D} \xrightarrow{\mathrm{TH(u)}} \mathrm{D} + \mathrm{m}, \qquad\qquad \mathrm{m} \xrightarrow{\delta_\mathrm{m}} \varnothing. \tag{1}$$

mRNA translation is initiated by free ribosome R binding with mRNA to form a translation initiation complex. Once the translation initiation complex is formed, we model elongation as a one step process that produces the protein. Since an mRNA co-transcript contains two independent ribosome binding sites and two stop codons, we model translation of the co-transcript as two independent translation events, where the mRNA can bind with ribosome with two different affinities to form two different translation initiation complexes:

$$\mathrm{m} + \mathrm{r} \underset{\kappa_\mathrm{p}^-}{\overset{\kappa_\mathrm{p}^+}{\rightleftharpoons}} \mathrm{C_p}, \qquad \mathrm{m} + \mathrm{r} \underset{\kappa_\mathrm{y}^-}{\overset{\kappa_\mathrm{y}^+}{\rightleftharpoons}} \mathrm{C_y}, \qquad \mathrm{C_p} \xrightarrow{\alpha} \mathrm{C_p} + \mathrm{p}, \qquad \mathrm{C_y} \xrightarrow{\alpha} \mathrm{C_y} + \mathrm{y}, \tag{2}$$

where r is free ribosome, C_p (C_y) is the translation initiation complex formed at the RBS of ECF32 (GFP), and α is the constant elongation rate constant. We assume that both proteins p and y decay with the same rate constant γ:

$$\mathrm{p}, \mathrm{y} \xrightarrow{\gamma} \varnothing. \tag{3}$$

The ECF32 binds with its cognate promoter pECF32 on a separate plasmid to transcribe sRNA-A. Let D_s represent the pECF32 promoter and let C_s represent the transcription initiation complex formed by ECF32 binding with pECF, we have the following reactions:

$$\mathrm{p} + \mathrm{D_s} \underset{\mathrm{k_s^-}}{\overset{\mathrm{k_s^+}}{\rightleftharpoons}} \mathrm{C_s}, \qquad \mathrm{C_s} \xrightarrow{\phi} \mathrm{C_s} + \mathrm{s}, \qquad \mathrm{s} \xrightarrow{\delta_s} \varnothing, \tag{4}$$

where s is sRNA-A, ϕ is its transcription rate constant proportional to the strength of the inducible promoter, and δ_s is the decay rate constant of the uncoupled sRNA-A. sRNA-A binds with the mRNA co-transcript (m_p and m_y) to form a RNA-complex c that is degraded rapidly with rate constant λ:

$$\mathrm{s} + \mathrm{m} \underset{\beta^-}{\overset{\beta^+}{\rightleftharpoons}} \mathrm{c}, \qquad \mathrm{c} \xrightarrow{\lambda} \varnothing. \tag{5}$$

By mass action kinetics, the ODE model of the reactions in (1)-(5) is:

$$\frac{\mathrm{d}}{\mathrm{d}t} C_p = \kappa_p^+ m r - \kappa_p^- C_p, \tag{6a}$$
$$\frac{\mathrm{d}}{\mathrm{d}t} C_y = \kappa_y^+ m r - \kappa_y^- C_y, \tag{6b}$$
$$\frac{\mathrm{d}}{\mathrm{d}t} C_s = k_s^+ p D_s - k_s^- C_s, \tag{6c}$$
$$\frac{\mathrm{d}}{\mathrm{d}t} c = \beta^+ m s - \beta^- c - \lambda c, \tag{6d}$$
$$\frac{\mathrm{d}}{\mathrm{d}t} m = TDH(u) - \delta_m m - \kappa_p^+ m r + \kappa_p^- C_p - \kappa_y^+ m r + \kappa_y^- C_y - \beta^+ m s + \beta^- c, \tag{6e}$$
$$\frac{\mathrm{d}}{\mathrm{d}t} s = \phi C_s - \delta_s s - \beta^+ m s + \beta^- c, \tag{6f}$$
$$\frac{\mathrm{d}}{\mathrm{d}t} p = \alpha C_p - \gamma p - k_s^+ p D_s + k_s^- C_s, \tag{6g}$$
$$\frac{\mathrm{d}}{\mathrm{d}t} y = \alpha C_y - \gamma y. \tag{6h}$$

We set the time derivatives in Supplementary Equations (6a)-(6d) to quasi-steady state to obtain the following:

$$C_p = \frac{mr}{\kappa_p}, \qquad C_y = \frac{mr}{\kappa y}, \qquad C_s = \frac{pD_s}{k_s}, \qquad c = \frac{ms}{\beta}, \tag{7}$$

where

$$\kappa_p := \frac{\kappa_p^-}{\kappa_p^+}, \qquad \kappa_y := \frac{\kappa_y^-}{\kappa_y^+}, \qquad k_s := \frac{k_s^-}{k_s^+}, \qquad \beta := \frac{\beta^- + \lambda}{\beta^+}.$$

In particular, κ_p and κ_y are the respective dissociation constants of the ribosome binding with the RBS's of ECF32 and GFP and k_s is the dissociation constant of ECF32 binding with its cognate pECF32 promoter. Stronger binding affinity between two molecules corresponds to a smaller dissociation constant. Substituting the results in (7) into Supplementary Equations (6e)-(6h), we obtained

the following reduced order model:

$$\frac{\mathrm{d}}{\mathrm{d}t}m = TDH(u) - \delta_{\mathrm{m}}m - \lambda ms/\beta, \tag{8a}$$

$$\frac{\mathrm{d}}{\mathrm{d}t}s = T_{\mathrm{s}}p - \delta_{\mathrm{s}}s - \lambda ms/\beta, \tag{8b}$$

$$\frac{\mathrm{d}}{\mathrm{d}t}p = Rm/\kappa_{\mathrm{p}} - \gamma p, \tag{8c}$$

$$\frac{\mathrm{d}}{\mathrm{d}t}y = Rm/\kappa_{\mathrm{y}} - \gamma y, \tag{8d}$$

where we have defined the lumped parameters $T_{\mathrm{s}} := \phi D_{\mathrm{s}}/k_{\mathrm{s}}$, and $R := \alpha r$. We note that due to the bi-cistronic operon design, the translation rate of ECF32, Rm/κ_{p}, and the translation rate of GFP, Rm/κ_{y}, are proportional to each other. The proportionality constant is given by the feedback gain k, defined in equation (1) in the main text, since:

$$\frac{Rm/\kappa_{\mathrm{p}}}{Rm/\kappa_{\mathrm{y}}} = \frac{\kappa_{\mathrm{y}}}{\kappa_{\mathrm{p}}} = k, \tag{9}$$

We note that by (8c) and (8d), the concentration dynamics of ECF32 and GFP follow:

$$p(t) = p(0)e^{-\gamma t} + \eta\frac{R}{\kappa_{\mathrm{y}}}e^{-\gamma t}\int_0^t e^{\gamma\tau}m(\tau)\mathrm{d}\tau, \quad y(t) = y(0)e^{-\gamma t} + \frac{R}{\kappa_{\mathrm{y}}}e^{-\gamma t}\int_0^t e^{\gamma\tau}m(\tau)\mathrm{d}\tau. \tag{10}$$

At steady state, we have:

$$\lim_{t\to+\infty} p(t) = k\frac{R}{\kappa_{\mathrm{y}}}e^{-\gamma t}\int_0^t e^{\gamma\tau}m(\tau)\mathrm{d}\tau, \qquad \lim_{t\to+\infty} y(t) = \frac{R}{\kappa_{\mathrm{y}}}e^{-\gamma t}\int_0^t e^{\gamma\tau}m(\tau)\mathrm{d}\tau, \tag{11}$$

and therefore, at steady state, the concentration of ECF32 is proportional to that of GFP by the feedback gain k:

$$\lim_{t\to+\infty} p(t) = k \lim_{t\to+\infty} y(t). \tag{12}$$

Thus, if we start an experiment from steady state (i.e., $p(0) = ky(0)$), Supplementary Equation (10) can be re-written as:

$$p(t) = k[y(0)e^{-\gamma t} + \frac{R}{\kappa_{\mathrm{y}}}e^{-\gamma t}\int_0^t e^{\gamma\tau}m(\tau)\mathrm{d}\tau], \quad y(t) = y(0)e^{-\gamma t} + \frac{R}{\kappa_{\mathrm{y}}}e^{-\gamma t}\int_0^t e^{\gamma\tau}m(\tau)\mathrm{d}\tau. \tag{13}$$

From (13), we obtain

$$p(t) = ky(t), \tag{14}$$

which implies that in this situation, ECF32 concentration is not only proportional to GFP concentration by feedback gain k at steady state, but also dynamically. Therefore, if the initial condition is such that $p(0) = ky(0)$, which is always satisfied when an experiment starts from a steady state (e.g., from overnight pre-culture), ECF32 concentration can be regarded as a dynamic sensor of GFP concentration even though they are not co-translated. Substituting (14) into (8b), we can further simplify model (8) to

$$\frac{\mathrm{d}}{\mathrm{d}t}m = TDH(u) - \delta_{\mathrm{m}}m - \lambda ms/\beta, \quad \frac{\mathrm{d}}{\mathrm{d}t}s = kT_{\mathrm{s}}y - \delta_{\mathrm{s}}s - \lambda ms/\beta, \quad \frac{\mathrm{d}}{\mathrm{d}t}y = Rm/\kappa_{\mathrm{y}} - \gamma y. \tag{15}$$

Activation of the resource competitor leads to a drop in the free concentration of ribosomes r, resulting in a change in the parameter R. To model this effect, we introduce a bounded disturbance

$d \in [0, \bar{d}]$ to (15), where $\bar{d} < 1$, that reduces the magnitude of R in the presence of competitor activation. Specifically, this leads to (15) to become:

$$\begin{aligned}
\frac{\mathrm{d}}{\mathrm{d}t}m &= TDH(u) - \delta_{\mathrm{m}}m - \lambda ms/\beta, \\
\frac{\mathrm{d}}{\mathrm{d}t}s &= kT_{\mathrm{s}}y - \delta_{\mathrm{s}}s - \lambda ms/\beta, \\
\frac{\mathrm{d}}{\mathrm{d}t}y &= R(1-d)m/\kappa_{\mathrm{y}} - \gamma y,
\end{aligned} \tag{16}$$

which is consistent with equations (2)-(4) in the main text. Our derivation of (16) relies on the assumption that free pECF concentration (D_{s}) is a constant, and therefore, parameter T_{s} is a constant. In practice, a fixed amount of DNA plasmid with pECF32 (D_t) are present, and binding of ECF32 (p) with pECF (D_{s}) reduces the free concentration of pECF32 (D_{s}). This effect can be modeled by replacing the constant T_{s} by a Hill function of p. We will further demonstrate this nonlinear effect on controller performance through simulations in Supplementary Note 11, yet we will use the simplified model (16) to develop intuition about the role of various system parameters on controller performance. This simplified model (16) is a good approximation since we have experimentally verified that pECF32 is a weak promoter with large linear dynamic range (Supplementary Figure 5). We finally remark that due to (12), even if the the initial condition assumption $p(0) = ky(0)$ is not satisfied, (16) can be used to study the system's steady state behavior.

Supplementary Note 8

sRNA-mediated feedback as a quasi-integral controller

In this Supplementary Note, we study the location of the steady state in Supplementary Equation (16) to establish the link between the sRNA-mediated post-TX controller and QIC. The notion of QIC was defined in [11]:

Definition: Consider a dynamical system

$$\frac{\mathrm{d}}{\mathrm{d}t}x = f(x, u, d; \epsilon), \qquad y = h(x; \epsilon), \tag{17}$$

with state x, a constant reference input u, a constant disturbance input d, a parameter $0 < \epsilon \ll 1$ and an output y. Suppose that system (17) has a unique locally stable steady state $\bar{x}$ and that functions $f(\cdot)$ and $h(\cdot)$ are smooth in all their arguments, system (17) realizes *ϵ-quasi-integral control* in an admissible input set $\mathbb{U} \times \mathbb{D}$ if for all $(u, d) \in \mathbb{U} \times \mathbb{D}$, the system's steady state output $\bar{y}$ is such that

$$\lim_{\epsilon \to 0} \bar{y}(u, d, \epsilon) = \bar{y}(u, 0, 0). \tag{18}$$

Supplementary Equation (18) implies that the effects of a disturbance d on the steady state output of a QIC system can be arbitrarily mitigated by decreasing the parameter ϵ, resulting in the system to behave like an integral control system at steady state. General conditions on system dynamics to satisfy QIC have been provided in [11]. Here, we specialize in the sRNA-mediated post-TX controller model (16) to demonstrate that it can achieve quasi-integral control under certain parameter conditions.

We first demonstrate the system's ability to achieve QIC when the parameter conditions (I) $\delta_\mathrm{m}/\lambda \ll 1$, (II) $\delta_\mathrm{m}/T \ll 1$, and (III) $\delta_\mathrm{m}/(kT_\mathrm{s}) \ll 1$ are all satisfied and then provide practical means to enforce these conditions in experiments. In particular, when (I)-(III) are satisfied, we can write $\lambda = \delta_\mathrm{m}/\epsilon$, $T = \nu_1\delta_\mathrm{m}/\epsilon$, and $kT_\mathrm{s} = \nu_2\delta_\mathrm{m}/\epsilon$, with dimensionless parameters $\nu_1, \nu_2 > 0$ and $0 < \epsilon \ll 1$. Using this re-parameterization, model (16) becomes

$$\epsilon\frac{\mathrm{d}}{\mathrm{d}t}m = \delta_\mathrm{m}[\nu_1 DH(u) - \epsilon m - ms/\beta], \tag{19a}$$

$$\epsilon\frac{\mathrm{d}}{\mathrm{d}t}s = \delta_\mathrm{m}[\nu_2 y - \mu\epsilon s - ms/\beta], \tag{19b}$$

$$\frac{\mathrm{d}}{\mathrm{d}t}y = R(1-d)m/\kappa_\mathrm{y} - \gamma y, \tag{19c}$$

where we have defined $\mu := \delta_\mathrm{s}/\delta_\mathrm{m}$. To compute the steady state of (19), we set the time derivatives to 0 to obtain the following algebraic equations:

$$\nu_1 DH(u) - \epsilon m - ms/\beta = 0, \qquad \nu_2 y - \epsilon\mu s - ms/\beta = 0, \qquad R(1-d)m/\kappa_\mathrm{y} - \gamma y = 0. \tag{20}$$

To demonstrate that (19) can achieve QIC, we first compute the solution of (20) when $\epsilon = 0$ and then infer the steady state location for small $\epsilon > 0$ using Implicit Function Theorem (IFT) [12]. When $\epsilon = 0$, the solution (m^*, s^*, y^*) of (20) is

$$y^* = \frac{\nu_1 DH(u)}{\nu_2}, \qquad m^* = \frac{\gamma\nu_1 DH(u)\kappa_\mathrm{y}}{\nu_2 R(1-d)}, \qquad s^* = \frac{\nu_2 R(1-d)\beta}{\gamma\kappa_\mathrm{y}}. \tag{21}$$

Note that since y^* is not a function of the disturbance d, perfect adaptation to disturbance d is achieved at $\epsilon = 0$. Next, we apply the IFT [12] to verify that for ϵ sufficiently small (but non-zero), the solution $(\bar{m}, \bar{s}, \bar{y})$ to (20) is close to (m^*, s^*, y^*) computed in (21). This would result in the

system with ϵ sufficiently small to have small adaptation error. To this end, we first let $x := [m, s, y]$ and re-write Supplementary Equation (20) as

$$F(x,\epsilon) = \begin{bmatrix} \nu_1 DH(u) - \epsilon m - ms/\beta \\ \nu_2 y - \epsilon\mu s - ms/\beta \\ R(1-d)m/\kappa_{\mathrm{y}} - \gamma y \end{bmatrix} = 0, \tag{22}$$

and note that $F(x,\epsilon)$ is continuously differentiable in both of its arguments. We then compute

$$\left.\frac{\partial F}{\partial x}\right|_{x=(m^*,s^*,y^*),\epsilon=0} = \begin{bmatrix} -\beta s^* & -\beta m^* & 0 \\ -\beta s^* & -\beta m^* & \nu_2 \\ R(1-d)/\kappa_{\mathrm{y}} & 0 & -\gamma \end{bmatrix}. \tag{23}$$

to verify that

$$\det\left[\left.\frac{\partial F}{\partial x}\right|_{x=(m^*,s^*,y^*),\epsilon=0}\right] = -\beta\nu_2 R(1-d)m^*/\kappa_{\mathrm{y}} \neq 0.$$

This allows us to apply IFT and claim that the solution $\bar{x} = \bar{x}(\epsilon)$ to $F(x,\epsilon) = 0$ is continuously differentiable in ϵ in a small neighborhood of 0. In particular, we have

$$\bar{y} = \bar{y}(u,d,\epsilon) = y^*(u) + \mathcal{O}(\epsilon) = \frac{\nu_1 DH(u)}{\nu_2} + \mathcal{O}(\epsilon) = \frac{TDH(u)}{kT_{\mathrm{s}}} + \mathcal{O}(\epsilon),$$

and subsequently,

$$\lim_{\epsilon\to 0} \bar{y}(u,d,\epsilon) = \bar{y}(u,0,0),$$

which satisfies (18). Hence, when the parameter conditions in (I)-(III) are satisfied, the sRNA-mediated post-TX controller can achieve QIC provided that the steady state $(\bar{m},\bar{s},\bar{y})$ is stable. We will show in the next Supplementary Note that for all positive parameters (e.g., regardless of the feedback gain), the sRNA-mediated post-TX controller always has a unique, locally stable steady state. These results would together imply that if mRNA transcription, sRNA transcription and mRNA-sRNA degradation are all sufficiently fast compared to RNA decay, corresponding respectively to the parameter conditions $\delta_{\mathrm{m}}/T \ll 1$, $\delta_{\mathrm{m}}/(kT_{\mathrm{s}}) \ll 1$, and $\delta_{\mathrm{m}}/\lambda \ll 1$, then the sRNA-mediated post-TX controller can essentially reject variation in free ribosome concentration d as a disturbance.

Now we consider if parameter conditions (I)-(III) can be satisfied in practice. Condition (I) is readily satisfied for the sRNA-mediated feedback because decay rate constant of the sRNA-mRNA complex (λ) is much larger than that of the uncoupled mRNA and/or sRNA ($\delta_{\mathrm{m}}, \delta_{\mathrm{s}}$) [13, 14]. Condition (II) can be satisfied for a regulated TX device with a reasonably strong promoter. In particular, the mRNA/DNA ratio for the Ec-TTL-P109 promoter is close to 2^6 [15]. With reference to the chemical reactions in (1), for constitutive transcription of a gene with copy number D, we have mRNA concentration dynamics $\mathrm{d}m/\mathrm{d}t = TD - \delta_{\mathrm{m}} m$. Using this simple model and the mRNA/DNA ratio provided in [15], at steady state, we have $\delta_{\mathrm{m}}/T = D/m \approx 2^{-6} = 0.015$, and therefore condition (II) is satisfied. The weaker BBa_J23116 promoter we choose is $\sim$ 20x weaker than the Ec-TTL-P109 promoter according to our unregulated TX devices' experiments (see Figure 5 in main text or Figure 7-8). This implies that for the weaker BBa_J23116 promoter, $\delta_{\mathrm{m}}/T \approx 0.3$. Parameter condition (III) can be reached by either having a sufficiently large T_{s}, which is dictated by the pECF32 promoter activity and its plasmid copy number, or by employing a strong feedback gain k.

To evaluate the necessity of implementing a high feedback gain k, we first perform some order-of-magnitude estimates for $\delta_{\mathrm{m}}/T_{\mathrm{s}}$ based on experimental results. Recall from Supplementary Note 7 that the parameter T_{s} is defined as $T_{\mathrm{s}} := \phi D_{\mathrm{s}}/k_{\mathrm{s}}$, where ϕ is the transcription rate constant proportional to pECF32 promoter strength, D_{s} is the plasmid copy number encoding pECF32, and k_{s} is the dissociation constant between ECF32 and pECF32. Our experiments have indicated that pECF32 is a rather weak promoter (i.e., small ϕ). In particular, we have performed aTc induction

experiments (Supplementary Figure 5C) for pECF32 and find its GFP per OD level to be $\sim 10^5$ when maximally induced. This is 10x lower than the GFP we observed from the unregulated TX device driven by the constitutive Ec-TTL-P109 promoter (Supplementary Figure 7A) despite the fact that (a) GFP RBS in the unregulated TX device (Supplementary Figure 7A) is 6x weaker than the GFP RBS used to perform functional test of ECF32 (Supplementary Figure 5C) and (b) pECF32 and GFP are on ColE2 plasmid (~ 60 copy) in the functional test experiment while on p15A plasmid (~ 15 copy) in the unregulated device (compare Supplementary Figure 4 vs. Figure 2c in the main text). These information leads us to estimate that pECF32 is ~ 200x weaker than the Ec-TTL-P109 promoter, and thus about 10x weaker than the BBa_J23116 promoter. On the other hand, since no direct measurement of k_{s} has been found in the literature, we use $k_{\mathrm{s}} = 100$ nM, which is in the range of the dissociation constant for common TX regulators [16]. This would result in $D_{\mathrm{s}}/k_{\mathrm{s}} \sim 1$ for pECF32 encoded on ColE2 plasmid. Therefore, taken together, we estimate that $\delta_{\mathrm{m}}/T_{\mathrm{s}} \sim 3$, and as a consequence, to satisfy $\delta_{\mathrm{m}}/(kT_{\mathrm{s}}) \ll 1$, we must increase the feedback gain, which can be enforced by increasing ECF32 RBS strength. The parametric analysis here therefore establishes that by implementing an sRNA-mediated post-TX controller with (I) a strong enough promoter and (II) a high feedback gain, quasi-integral control can be realized. Since both promoters we use are strong enough according to our analysis, increasing the feedback gain by increasing ECF32 RBS strength improves a regulated TX device's robustness.

Finally, we clarify that, more generally, for a circuit with the same chemical reaction structure (but possibly different parameters and reactants), increasing the feedback gain may not always increase robustness. This is because the mathematical analysis we performed here only concludes that when mRNA transcription and mRNA-sRNA complex formation rates are both already sufficiently large, sRNA transcription rate is the major bottleneck to the feedback system's robustness and therefore adaption performance improves with increasing feedback gain, which effectively increases sRNA's transcription rate. Although general theories exist [11], in practice, a case-by-case parametric study is essential to identify the key tunable physical parameter that dictates robustness performance.

Supplementary Note 9

Stability of the sRNA-mediated post-TX controller

In this Note, we show the uniqueness and stability of the steady state of (16) independent of the parameter conditions (I)-(III). The steady state $(\bar{m}, \bar{s}, \bar{y})$ of (16) can be found by setting the time derivatives to zero:

$$0 = TDH(u) - \delta_{\rm m}\bar{m} - \theta\bar{m}\bar{s}, \qquad 0 = T_{\rm s}k\bar{y} - \delta_{\rm s}\bar{s} - \theta\bar{m}\bar{s}, \qquad R(1-d)\bar{m}/\kappa_{\rm y} = \gamma\bar{y}, \tag{24}$$

where for simplicity of notation we defined $\theta := \lambda/\beta$. Solution to (24) can be found by the following algebraic equations:

$$\bar{y} = \frac{R(1-d)\bar{m}}{\kappa_{\rm y}\gamma}, \quad \bar{s} = \frac{TDH(u) - \delta_{\rm m}\bar{m}}{\theta\bar{m}}, \tag{25}$$

$$\left[\frac{T_{\rm s}kR(1-d)}{\kappa_{\rm y}\gamma} + \delta_{\rm m}\right]\bar{m} - \frac{TDH(u)\delta_{\rm s}}{\theta\bar{m}} = -\frac{\delta_{\rm m}\delta_{\rm s}}{\theta} + TDH(u) \tag{26}$$

Since for all $\bar{m} > 0$, the left-hand side of Supplementary Equation (26) is monotonically increasing and the right-hand side of it is a constant, Supplementary Equation (26) has a unique positive solution $\bar{m} > 0$. Steady state of $\bar{s}$ and $\bar{y}$ can then be obtained uniquely from (25). We study the local stability of (16) by linearizing it around its unique steady state $(\bar{y}, \bar{m}, \bar{s})$ to obtain the following linearized system:

$$\frac{\rm d}{{\rm d}t}\begin{bmatrix} y \\ m \\ s \end{bmatrix} = \underbrace{\begin{bmatrix} -\gamma & R(1-d)/\kappa_{\rm y} & 0 \\ 0 & -\delta_{\rm m} - \theta\bar{s} & -\theta\bar{m} \\ T_{\rm s}k & -\theta\bar{s} & -\delta_{\rm s} - \theta\bar{m} \end{bmatrix}}_{A} \begin{bmatrix} y \\ m \\ s \end{bmatrix} \tag{27}$$

We study stability of (27) by applying Routh-Hurwitz stability criterion to the characteristic polynomial $\mathcal{P}(A)$ of matrix A:

$$\begin{aligned}\mathcal{P}(A) &= (s+\gamma)(s+\delta_{\rm m}+\theta\bar{s})(s+\delta_{\rm s}+\theta\bar{m}) + R(1-d)\theta\bar{m}T_{\rm s}k/\kappa_{\rm y} - \theta^2\bar{m}\bar{s}(s+\gamma) \\ &= s^3 + [\gamma+\delta_{\rm s}+\delta_{\rm m}+\theta(\bar{m}+\bar{s})]s^2 + [\theta\bar{m}(\gamma+\delta_{\rm m}) + \theta\bar{s}(\gamma+\delta_{\rm s}) + \gamma(\delta_{\rm m}+\delta_{\rm s}) + \delta_{\rm m}\delta_{\rm s}]s \\ &\quad + R(1-d)\theta\bar{m}T_{\rm s}k/\kappa_{\rm y} + \gamma\theta(\bar{s}\delta_{\rm s} + \bar{m}\delta_{\rm m}) + \gamma\delta_{\rm m}\delta_{\rm s}.\end{aligned} \tag{28}$$

By Routh-Hurwitz stability criterion, system (27) is stable if and only if

$$\begin{aligned}\mathcal{L} := [\gamma+\delta_{\rm s}+\delta_{\rm m}+\theta(\bar{m}+\bar{s})]\cdot[\theta\bar{m}(\gamma+\delta_{\rm m}) + \theta\bar{s}(\gamma+\delta_{\rm s}) + \gamma(\delta_{\rm m}+\delta_{\rm s}) + \delta_{\rm m}\delta_{\rm s}] > \\ \mathcal{R} := R(1-d)\theta\bar{m}T_{\rm s}k/\kappa_{\rm y} + \gamma\theta(\bar{s}\delta_{\rm s} + \bar{m}\delta_{\rm m}) + \gamma\delta_{\rm m}\delta_{\rm s}.\end{aligned} \tag{29}$$

On the one hand, note that since $\bar{m},\ \bar{s} > 0$ and $\delta_{\rm s},\ \delta_{\rm m},\ \gamma,\ \theta > 0$, we have

$$\begin{aligned}\mathcal{L} &= [\gamma+\delta_{\rm s}+\delta_{\rm m}+\theta(\bar{m}+\bar{s})]\cdot[\theta\bar{m}(\gamma+\delta_{\rm m}) + \theta\bar{s}(\gamma+\delta_{\rm s}) + \gamma(\delta_{\rm m}+\delta_{\rm s}) + \delta_{\rm m}\delta_{\rm s}] \\ &> \theta\gamma\delta_{\rm s}\bar{s} + \theta^2\gamma\bar{m}\bar{s} + \gamma\theta(\delta_{\rm s}\bar{s} + \delta_{\rm m}\bar{m}) + \gamma\delta_{\rm m}\delta_{\rm s}.\end{aligned} \tag{30}$$

On the other hand,

$$\begin{aligned}\mathcal{R} &= R(1-d)\theta\bar{m}T_{\rm s}k/\kappa_{\rm y} + \gamma\theta(\bar{s}\delta_{\rm s} + \bar{m}\delta_{\rm m}) + \gamma\delta_{\rm m}\delta_{\rm s} \\ &= \gamma TDH(u)\delta_{\rm s}/\bar{m} - \gamma\delta_{\rm m}\delta_{\rm s} + \gamma\theta TDH(u) - \gamma\theta\delta_{\rm m}\bar{m} + \gamma\theta(\bar{s}\delta_{\rm s} + \bar{m}\delta_{\rm m}) + \gamma\delta_{\rm m}\delta_{\rm s} \qquad (31) \\ &< \theta\gamma\delta_{\rm s}\bar{s} + \theta^2\gamma\bar{m}\bar{s} + \gamma\theta(\bar{s}\delta_{\rm s} + \bar{m}\delta_{\rm m}) + \gamma\delta_{\rm m}\delta_{\rm s}, \qquad (32)\end{aligned}$$

where we have substituted in (26) to derive (31) and substituted in (25) to derive (32). Comparing (30) and (32), we have $\mathcal{L} > \mathcal{R}$ and hence (29) is verified. Therefore, the positive steady state $(\bar{m}, \bar{s}, \bar{y})$ is unique and locally stable for any positive parameters $T, D, H(u), T_{\rm s}, k, \delta_{\rm s}, \delta_{\rm m}, \theta, \kappa_{\rm y}$ and $R(1-d)$.

Supplementary Note 10

A model of the sRNA silencing circuit

In Figure 4 of the main text, we constructed a circuit to test the sRNA silencing and the ECF32's actuating mechanisms. Here, we describe a simple mathematical model to simulate the dose response curve of this circuit. In fact, a model of this circuit is readily available by slight modification of equations (2)-(4) in the main text (or equivalently, Supplementary Equation (15)), which are used to describe a regulated TX device. This is because the only difference between an sRNA silencing circuit (Figure 4a) and a regulated TX device (Figure 2a) is that sRNA transcription in the former is regulated by active TetR, while it is regulated by ECF32 in the latter. We therefore study a model for the sRNA silencing circuit:

$$\frac{\mathrm{d}}{\mathrm{d}t}m = TD - \delta_{\mathrm{m}}m - \theta ms, \qquad \frac{\mathrm{d}}{\mathrm{d}t}s = T_{\mathrm{s}}H_{\mathrm{s}}(v) - \delta_{\mathrm{s}}s - \theta ms, \qquad \frac{\mathrm{d}}{\mathrm{d}t}y = Rm/\kappa_{\mathrm{y}} - \gamma y. \tag{33}$$

All parameters are defined identically as in Supplementary Note 7, except that we have introduced $H_{\mathrm{s}}(v)$ to describe activation of sRNA transcription through the addition of TetR's effector aTc, whose concentration is represented by v. In particular, since TetR is a dimer [17], we have

$$H_{\mathrm{s}}(v) = \frac{(v/k_d)^2}{1 + (v/k_d)^2}, \tag{34}$$

where k_d is an effective dissociation constant that describes the binding between aTc and TetR and between TetR and the pTet promoter. This is based on the assumption that a sufficiently large amount of TetR has been produced, so the active amount of TetR depends solely on aTc concentration (v). Simulation of (33) is shown in Supplementary Figure 15.

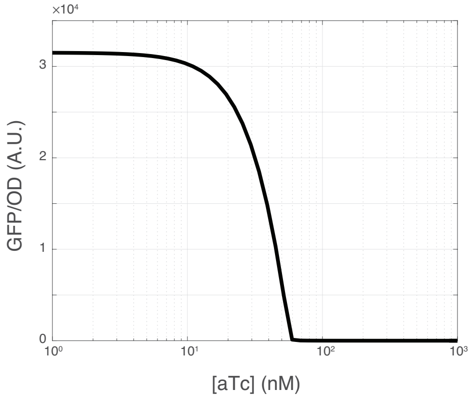

Supplementary Figure 15: Simulation of the sRNA silencing model in Supplementary Equation (33). Simulation parameters: $k_{\mathrm{d}} = 100$ nM, $\delta_{\mathrm{m}} = \delta_{\mathrm{s}} = 1$ hr^{-1}, $T_{\mathrm{s}} = 2000$ nM·hr^{-1}, $TD = 500$ nM·hr^{-1}, $\gamma = 1$ hr^{-1}, $\theta = 10$ nM^{-1}·hr^{-1}, $R/\kappa_{\mathrm{y}} = 0.63$ hr^{-1}, and 1 nM converts to 10^3 A.U..

Supplementary Note 11

pECF Promoter saturation

In the development of model (16), we assumed that the free concentration of pECF32 (D_s) is a constant, resulting in the transcription rate of sRNA to be $T_\mathrm{s}p = T_\mathrm{s}ky$, which is proportional to the concentrations of GFP y and ECF32 p. While this linearity assumption simplifies understanding of the feedback and the role of the feedback gain k, transcription of sRNA is a nonlinear function of p in practice. In particular, if p is too large to saturate the pECF32 promoter, sRNA transcription becomes unresponsive to changes in sensor concentration, breaking the feedback loop. We have characterized pECF32 in Supplementary Figure 4C, where the expression of *sfGFP* gene driven by pECF32 increases with aTc induction even for the maximum amount of aTc applied (1000 nM). This implies that pECF32 has a wide dynamic range (i.e., large dissociation constant k_s) and is therefore less likely to be saturated. Nevertheless, in this Supplementary Note, we provide a model taking promoter saturation into account and use it to perform numerical simulations and to understand the constraint promoter saturation imposes on controller design.

Let D_s^t and D_s be the total and the free concentrations of DNA encoding sRNA, respectively, the following conservation law applies [16, 18]:

$$D_\mathrm{s}^t = D_\mathrm{s} + C_\mathrm{s}, \qquad \Rightarrow \qquad D_\mathrm{s} = \frac{D_\mathrm{s}^t}{1 + p/k_\mathrm{s}}. \tag{35}$$

Therefore, we modify (8) to become:

$$\frac{\mathrm{d}}{\mathrm{d}t}m = TDH(u) - \delta_\mathrm{m}m - \lambda ms/\beta, \tag{36a}$$

$$\frac{\mathrm{d}}{\mathrm{d}t}s = \tilde{T}_\mathrm{s}\frac{p/k_\mathrm{s}}{1 + p/k_\mathrm{s}} - \delta_\mathrm{s}s - \lambda ms/\beta, \tag{36b}$$

$$\frac{\mathrm{d}}{\mathrm{d}t}p = Rm/\kappa_\mathrm{p} - \gamma p, \tag{36c}$$

$$\frac{\mathrm{d}}{\mathrm{d}t}y = Rm/\kappa_\mathrm{y} - \gamma y, \tag{36d}$$

where $\tilde{T}_\mathrm{s} := \phi D_\mathrm{s}^t$. The only difference between (36) and (8) is that in (36b), sRNA transcription rate is a Hill function of p and may become saturated if p/k_s is large. In contrast, if $p/k_\mathrm{s} \ll 1$, we have

$$\frac{\mathrm{d}}{\mathrm{d}t}s = \tilde{T}_\mathrm{s}p/k_\mathrm{s} - \delta_\mathrm{s}s - \lambda ms/\beta,$$

which is in the same form as (8b). Similarly, following the reasoning in (13)-(14), we can re-write (36) as:

$$\begin{aligned}
\frac{\mathrm{d}}{\mathrm{d}t}m &= TDH(u) - \delta_\mathrm{m}m - \lambda ms/\beta,\\
\frac{\mathrm{d}}{\mathrm{d}t}s &= \tilde{T}_\mathrm{s}\frac{ky/k_\mathrm{s}}{1 + ky/k_\mathrm{s}} - \delta_\mathrm{s}s - \lambda ms/\beta,\\
\frac{\mathrm{d}}{\mathrm{d}t}y &= R(1-d)m/\kappa_\mathrm{y} - \gamma y.
\end{aligned} \tag{37}$$

Note that from (37), since k and k_s are always lumped together, increasing feedback gain k is effectively increasing the pECF32 promoter strength (i.e., decreasing $k_\mathrm{s}^\mathrm{eff} := k_\mathrm{s}/k$). Since directly engineering the interaction between the ECF sigma factor and its cognate promoter requires a significant amount of efforts, the feedback gain k becomes a convenient and effective approach to provide tunability to this parameter. In [11], we have shown that system (37) contains a unique, locally

stable, positive steady state, and derivation of this result is similar to the one in Supplementary Note 9.

When designing a regulated device with a desired level of nominal output, factoring pECF promoter saturation into the model leads to additional design considerations, especially when the pECF promoter is strong. In general, for a chosen ECF sigma factor and its cognate promoter, the following two design strategies can lead to two TX devices with identical nominal outputs but different robustness performances:

- Design I: a regulated TX device with stronger promoter of the regulated gene and high feedback gain,
- Design II: a regulated TX device with weaker promoter of the regulated gene and some lower feedback gain (to match the nominal output level of design I).

In Figures 5-6 of the main text, for ECF32, our experiments suggest that Design I is more robust than Design II and therefore is more preferable. This result is consistent with our model analysis in Supplementary Note 8, where, in addition to the standing assumptions that mRNA-sRNA interaction and mRNA transcription are sufficiently fast, we assume that the pECF promoter is not saturated when operating in a closed loop configuration. Our experimental and numerical results support this assumption for pECF32. Under this assumption, we produce a similar result in Supplementary Figure 16A by simulating equation (16) with a different set of parameters listed in the figure caption.

By contrast, if the pECF promoter in a regulated device is saturated, a decrease in ECF concentration arising from a drop in ribosome availability does not lead to decreased pECF promoter activity and subsequently sRNA transcription. This breaks the feedback loop, causing the regulated TX device to lose its ability to adapt to ribosome availability changes. Compared to Design II, the pECF promoter in Design I is more likely to become saturated because they have the same nominal outputs and the larger feedback gain in Design I leads to a higher ECF protein concentration and, consequently, high occupancy of the pECF promoter.

Therefore, given the same nominal outputs, when comparing the robustness of Design I and II with pECF promoter saturation taken into account, Design I is not necessarily more robust than Design II. This is exemplified in Supplementary Figure 16B, where we simulated equation (37), and found that Design II (black line with circles) is more robust than Design I (red line with squares) for some low levels of nominal outputs. The fact that the nominal output for Design I with the highest feedback gain (dark red square) in Supplementary Figure 16B is much higher than that in Supplementary Figure 16A, where promoter saturation is not included in the model, implies that the pECF promoter in Supplementary Figure 16B is saturated by ECF proteins and could not responsively activate sRNA to further repress the regulated gene's expression. This subsequently leads to reduced robustness performance. These observations imply that when choosing parts to construct sRNA-mediated regulated devices with a desired nominal output, for a fixed pECF promoter, it is important to balance (i) the requirement to increase sRNA transcription rate by increasing feedback gain to reduce integration leakiness for improved adaptation (see Supplementary Note 8) and (ii) the requirement to avoid pECF promoter saturation by reducing feedback gain. In particular, if the pECF promoter is weak, integration leakiness is the main robustness bottleneck due to slow sRNA transcription and properly increasing feedback gain can lead to improved robustness performance without triggering pECF promoter saturation. By contrast, if one uses a strong pECF promoter and sRNA transcription is already fast enough, the main design consideration is to avoid pECF promoter saturation. In this case, a low feedback gain is likely to be sufficient.

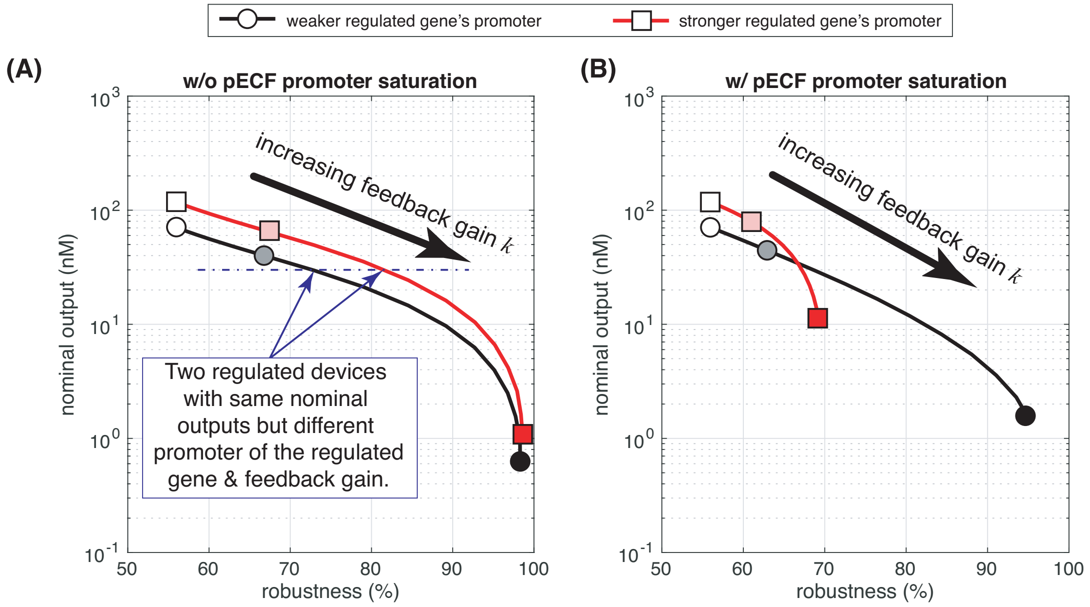

Supplementary Figure 16: Effect of pECF promoter saturation on robustness of two regulated TX design strategies. (A) Simulation of model (16), where pECF promoter saturation is not included (i.e., constant free pECF promoter concentration). (B) Simulation of model (37) where pECF promoter saturation is included. Identical to Figure 5 in the main text, nominal output is defined as the output protein's concentration in the absence of disturbance and robustness is defined as (nominal output)/(output in the presence disturbance) $\times 100\%$. A disturbance of $d = 50\%$ drop in translation rate is used in all simulations to evaluate robustness. Illustrated square and circle markers correspond to performances of circuits with feedback gains $k = 0.01$, 0.78 and 100, respectively, with gain values reflected by the darkness of color fill of the markers. Blue arrow points to two example points in the performance space for comparison of two regulated devices discussed in Supplementary Note 11. Simulation in panel B is performed using (37) with parameters: $TDH(u) = 60$ nM$\cdot$hr^{-1} (weaker regulated gene's promoter), $TDH(u) = 100$ nM$\cdot$hr^{-1} (stronger regulated gene's promoter), $\tilde{T}_{\rm s} = 100$ nM$\cdot$hr^{-1}, $k_{\rm s} = 100$ nM, $\gamma = 0.5$ hr^{-1}, $\delta_{\rm m} = \delta_{\rm s} = 3$ hr^{-1}, $\theta = 10^3$ nM^{-1}hr^{-1}, $R = 10$ μM$\cdot$hr^{-1} and $\kappa_{\rm y} = 5\mu$M. For panel A, we used model (16) and set $T = \tilde{T}/k_{\rm s}$, with other parameters identical to those in panel B.

Supplementary Note 12

Increasing ribosome availability may not mitigate ribosome competition effects

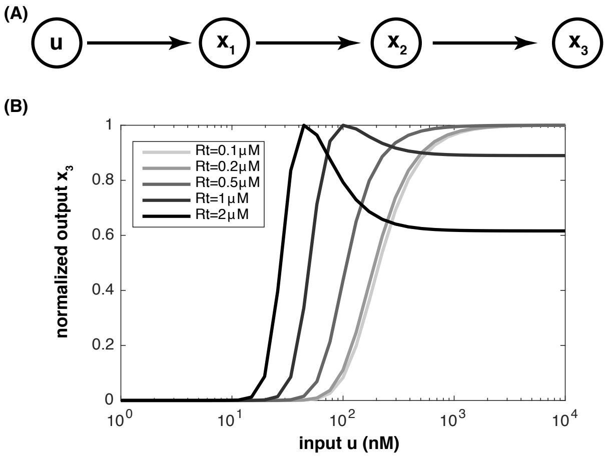

Supplementary Figure 17: (A) Three-stage activation cascade studied in Supplementary Note 12. (B) Increasing the total amount of ribosomes may change the dose response curve of the activation cascade from monotonically increasing to biphasic. This implies that increasing the total amount of ribosomes may not help mitigate unintended interactions arising from ribosome competition. Simulation parameters: $T_1 = T_3 = 100$ nM·hr^{-1}, $T_2 = 500$ nM·hr^{-1}, $\gamma = 0.5$ hr^{-1}, $\delta = 2$ hr^{-1}, $\kappa_1 = \kappa_3 = 1000$ nM, $\kappa_2 = 100$ nM, $n_i = 2$, $k_1 = k_2 = 100$ nM, $k_3 = 200$ nM. The total ribosome amount is indicated in the legend.

This note is intended to study the effectiveness of a centralized approach to mitigate the unintended effects arising from ribosome competition. We take the transcriptional activation cascade composed of three TX devices in Supplementary Figure 17 as an example. We have shown experimentally in [19] that the dose response curve of a two-stage activation cascade can be biphasic due to resource competition. We follow the general mathematical model derived in [19] and write the model for the three-stage activation cascade as:

$$\frac{\mathrm{d}}{\mathrm{d}t}m_i = T_i F_i(u_i) - \delta m_i, \qquad \frac{\mathrm{d}}{\mathrm{d}t}x_i = R m_i/\kappa_i - \gamma x_i, \tag{38}$$

where $i = 1, 2, 3$; T_i is the maximum transcription rate constant; δ and γ are respectively the decay rate constant of the mRNA and the protein; κ_i is the ribosome binding site strength of device i; and R is the free amount of ribosomes; Function $F_i(\cdot)$ describes transcriptional regulation of device i by its input u_i. For the activation cascade, we have $u_i = x_{i-1}$ $(i = 2, 3)$, $u_1 = u$, and

$$F_i(u_i) = \frac{(u_i/k_i)^{n_i}}{1 + (u_i/k_i)^{n_i}}, \tag{39}$$

where k_i is the dissociation constant of u_i binding with the promoter in device i and n_i is the cooperativity of the binding. To model resource-induced interactions, we use the following conservation law for the ribosomes:

$$R_t = R(1 + \sum_{i=1}^{3} m_i/\kappa_i). \tag{40}$$

When using a centralized approach as in [20, 21], additional ribosomes or orthogonal ribosomes are re-allocated to the synthetic circuit in an effort to mitigate ribosome competition effect. We therefore model it as an increase in the total amount of ribosomes (R_t). In Supplementary Figure 17B, we simulated (38)-(40) to obtain the dose response of the activation cascade with different levels of R_t. With the baseline total ribosome concentration of $R_t = 100$), the dose response curve of the activation cascade is monotonically increasing as intended. However, when total ribosome concentration increases, the dose response curve progressively becomes biphasic.

This phenomenon can be explained by the following ribosome competition mechanism. When total ribosome concentration (R_t) increases, an increased amount of protein x_1, the first protein in the TX cascade, is produced due to an increase in its TL rate. Since x_1 is a TX activator of x_2, the second protein in the TX cascade, an increase in x_1 concentration promotes TX of x_2's mRNA. The increase in x_2's mRNA leads to an increased ribosome demand. If x_2's mRNA employs a strong RBS, a significant amount of ribosomes will be bound to x_2's mRNA, which, in turn, reduces the free amount of ribosomes. As a result, the TL rate of output x_3 may decrease when the total amount of ribosome has increased. Under certain parameter conditions, this effect makes the output x_3 to decrease progressively with increasing total ribosome concentration, rendering the dose response curve to become biphasic. This simulation result suggests that in certain situations a centralized (i.e., increase ribosome availability) may not be sufficient to mitigate the unintended effects of ribosome competition.

Supplementary Note 13

Numerical Simulation

Simulations were carried out using MATLAB R2015b with variable step ODE solver `ode23s` for equations (2)-(4) in the main text. Simulation parameters were taken from a set of characteristic values from literature for bacterium *E. coli* and listed below in Supplementary Table 8. Based on these characteristic values, the parameters are chosen to qualitatively match the experimental results in Figure 5a of the main text, with specific focus to reflect the following observations:

- $\sim$ 20 fold difference in nominal outputs between high-gain regulated and low-gain regulated devices driven by the stronger promoter;
- similar nominal output levels for the high-gain regulated device driven by the stronger promoter and the high-gain unregulated device driven by the weaker promoter;
- at least 90% robustness for the high-gain regulated device driven by the stronger promoter and reduced robustness for the high-gain regulated device driven by the weaker promoter;
- the order-of-magnitude parameter estimation performed in Supplementary Note 8 for the TX of mRNA and sRNA.

The chosen parameters used to generate Figures 3a-b and 5a of the main text are listed in Supplementary Table 9. In particular, Figure 3a was simulated using the strong promoter parameter (i.e., $TD = 200$ nM$\cdot$hr^{-1}). Figure 3b was simulated using the parameters for strong promoter and high gain (i.e., $TD = 200$ nM$\cdot$hr^{-1} and $k = 4$). When simulating unregulated devices, the lumped transcription rate constant of sRNA T_s is set to 0. The other parameters remain unchanged for all simulations. Simulation of (I) steady state performance and (II) temporal response to step decrease in ribosome availability for all TX devices using the parameters listed in Supplementary Table 9 are provided in Supplementary Figure 18.

Parameters		Characteristic values	Sources
DNA copy number	-	1 copy $\sim$ 1 nM	[22]
Uncoupled RNA decay rate constant	δ_m	$3 \sim 20$ hr^{-1}	[23, 24][1]
Protein dilution rate constant	γ	$0.4 - 0.7$ hr^{-1}	experiment
mRNA-sRNA annihilation rate constant	θ	~ 100 nM$^{-1}\cdot$hr^{-1}	[14, 25]
Maximum translation rate constant	R	~ 100 μM$\cdot$hr^{-1}	[5, 9, 19][2]
Dis. const. between ribosome and RBS	κ	$\gtrsim 1$ μM	[5, 19]

Supplementary Table 8: Characteristic parameter values in bacterium *E. coli*

[1] Corresponding to half life between 2 and 13 mins.

[2] Based on total ribosome concentration of 1 μM and translation rate constant of 100 hr^{-1}.

Parameter	Value	Unit	Remark
TD	[15, 200]	nM·hr^{-1}	For the weaker and the stronger promoters, respectively.
$H(u_1)$	1	-	Constitutive promoter.
T_s	0.8	hr^{-1}	See discussion in Supplementary Note 8.
δ_m	5	hr^{-1}	-
δ_s	5	hr^{-1}	-
γ	0.5	hr^{-1}	Experiment
θ	100	nM^{-1}·hr^{-1}	-
R	100	μM·hr^{-1}	-
κ_y	10	μM	-
k	[0.05,0.6,4]	-	For low, medium and high feedback gains, respectively.
d	[0,25%,50%]		To model [0,6,1000] nM AHL induction.

Supplementary Table 9: Simulation parameters

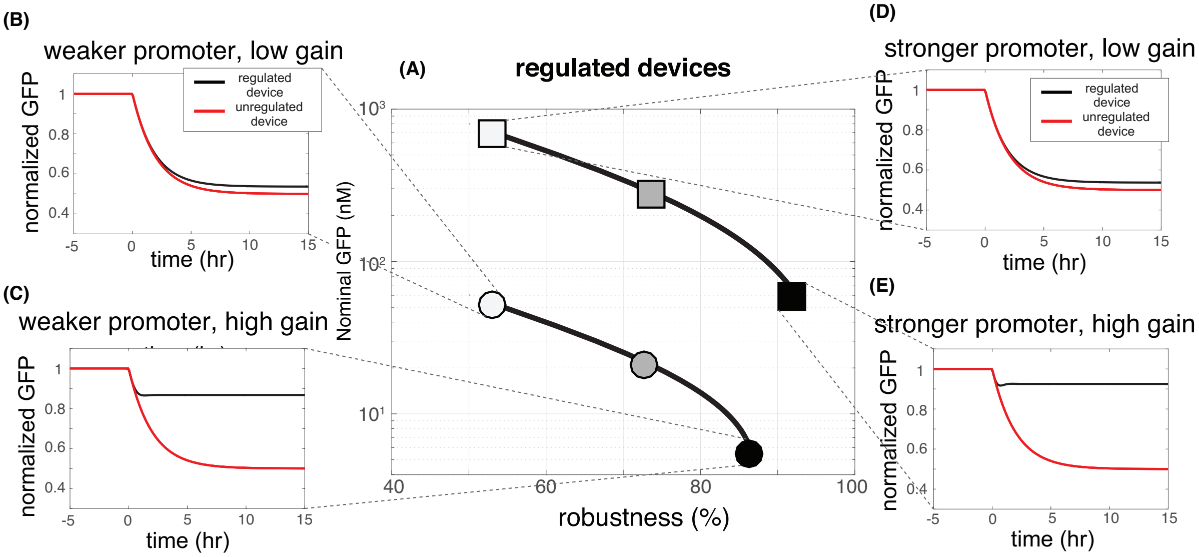

Supplementary Figure 18: Simulation of TX devices using equations (2)-(4) in main text and parameters listed in Supplementary Table 9. (A) Robustness and nominal output of the regulated TX devices. Robustness = (GFP when $d = 50\%$) × 100%/(GFP when $d = 0$) and nominal GFP = GFP when $d = 0$. Symbols □ and ◯ each represent regulated devices with the stronger or the weaker promoter. A symbol's color filling in gray scale from light to dark represents a feedback gain from low to high. (B)-(E) Temporal responses of 4 regulated and their unregulated counterparts when subject to a d =50% disturbance injected at $t = 0$.

Supplementary References

[1] Joshua T. Kittleson, Sherine Cheung, and J. Christopher Anderson. Rapid optimization of gene dosage in *E. coli* using DIAL strains. *J. Biol. Eng.*, 5(1):10, 2011.

[2] Howard M. Salis, Ethan A. Mirsky, and Christopher A. Voigt. Automated design of synthetic ribosome binding sites to control protein expression. *Nat. Biotechnol.*, 27(10):946–50, 2009.

[3] Virgil A. Rhodius, Thomas H. Segall-Shapiro, Brain D. Sharon, Amar Ghodasara, Ekaterina Orlova, Hannah Tabakh, David H. Burkhardt, Kevin Clancy, Todd C. Peterson, Carol A. Gross, and Christopher A. Voigt. Design of orthogonal genetic switches based on a crosstalk map of σs, anti-σs, and promoters. *Mol. Syst. Biol.*, 9(1):702–702, 2013.

[4] Tian Tian and Howard M. Salis. A predictive biophysical model of translational coupling to coordinate and control protein expression in bacterial operons. *Nucleic Acids Res.*, 43(14):7137–7151, 2015.

[5] Andras Gyorgy, José I. Jiménez, John Yazbek, Hsin-Ho Huang, Hattie Chung, Ron Weiss, and Domitilla Del Vecchio. Isocost lines describe the cellular economy of genetic circuits. *Biophys. J.*, 109(3):639–646, 2015.

[6] Stephen A. Bustin, Vladimir Benes, Jeremy A. Garson, Jan Hellemans, Jim Huggett, Mikael Kubista, Reinhold Mueller, Tania Nolan, Michael W. Pfaffl, Gregory L. Shipley, Jo Vandesompele, and Carl T. Wittwer. The MIQE Guidelines: *M*inimum *I*nformation for publication of *Q*uantitative real-time PCR *E*xperiments. *Clin. Chem.*, 55(4):611–622, 2009.

[7] Kang Zhou, Lihan Zhou, Qing 'En Lim, Ruiyang Zou, Gregory Stephanopoulos, and Heng-Phon Too. Novel reference genes for quantifying transcriptional responses of *Escherichia coli* to protein overexpression by quantitative PCR. *BMC Mol. Biol.*, 12(1):18, 2011.

[8] Jesper Vind, Michael A. Sørensen, Michael D. Rasmussen, and Steen Pedersen. Synthesis of proteins in Escherichia coli is limited by the concentration of free ribosomes: Expression from reporter gene does not always reflect functional mRNA levels. *J. Mol. Biol.*, 231:678–688, 1993.

[9] Hans Bremer and Patrick P. Dennis. Modulation of chemical composition and other parameters of the cell by growth rate. In Frederick C. Neidhardt, editor, *Escherichia coli and Salmonella: Cellular and Molecular Biology*. ASM Press, 1996.

[10] Francesca Ceroni, Rhys Algar, Guy-Bart Stan, and Tom Ellis. Quantifying cellular capacity identifies gene expression designs with reduced burden. *Nat. Methods*, 12(5):415–422, 2015.

[11] Yili Qian and Domitilla Del Vecchio. Realizing 'integral control' in living cells: how to overcome leaky integration due to dilution? *J. R. Soc. Interface.*, 15(139):20170902, 2018.

[12] Walter Rudin. *Principles of Mathematical Analysis*. McGraw-Hill, 3rd edition, 1976.

[13] Erel Levine, Zhongge Zhang, Thomas Kuhlman, and Terence Hwa. Quantitative characteristics of gene regulation by small RNA. *PLoS Biol.*, 5(9):e229, 2007.

[14] Razika Hussein and Han N. Lim. Direct comparison of small RNA and transcription factor signaling. *Nucleic Acids Res.*, 40(15):7269–7279, 2012.

[15] Sriram Kosuri, Daniel B. Goodman, Guillaume Cambray, Vivek K. Mutalik, Yuan Gao, Adam P. Arkin, Drew Endy, and George M. Church. Composability of regulatory sequences controlling transcription and translation in *Escherichia coli*. *Proc. Natl. Acad. Sci. U. S. A.*, 110(34):14024–14029, 2013.

[16] Domitilla Del Vecchio and Richard M. Murray. *Biomolecular Feedback Systems*. Princeton University Press, Princeton, 2014.

[17] Sara Hooshangi, Stephan Thiberge, and Ron Weiss. Ultrasensitivity and noise propagation in a synthetic transcriptional cascade. *Proc. Natl. Acad. Sci. U. S. A.*, 102(10):3584–3586, 2005.

[18] Uri Alon. *An Introduction to Systems Biology: Design Principles of Biological Circuits.* Chapman & Hall/CRC Press, 2006.

[19] Yili Qian, Hsin-Ho Huang, José I. Jiménez, and Domitilla Del Vecchio. Resource competition shapes the response of genetic circuits. *ACS Synth. Biol.*, 6(7):1263–1272, 2017.

[20] Alexander P. S. Darlington, Juhyun Kim, José I. Jiménez, and Declan G. Bates. Dynamic allocation of orthogonal ribosomes facilitates uncoupling of co-expressed genes. *Nat. Commun.*, 9(1), 2018.

[21] Ophelia S. Venturelli, Mika Tei, Stefan Bauer, Leanne Jade G. Chan, Christopher J. Petzold, and Adam P Arkin. Programming mRNA decay to modulate synthetic circuit resource allocation. *Nat. Commun.*, 8:15128, 2017.

[22] Ron Milo and Rob Phillips. *Cell Biology by the Numbers.* Garland Science, 2015.

[23] Jonathan A. Bernstein, Pei-Hsun Lin, Stanley N. Cohen, and Sue Lin-Chao. Global analysis of *Escherichia coli* RNA degradosome function using DNA microarrays. *Proc. Natl. Acad. Sci. U. S. A.*, 101(9):2758–2763, 2004.

[24] Necmettin Yildirim and Michael C. Mackey. Feedback regulation in the lactose operon: A mathematical modeling study and comparison with experimental data. *Biophys. J.*, 84(5):2841–2851, 2003.

[25] Aurlie Fender, Johan Elf, Kornelia Hampel, Bastian Zimmermann, and E. Gerhart H. Wagner. RNAs actively cycle on the Sm-like protein Hfq. *Genes Dev.*, 24(23):2621–2626, 2010.
