# Supplementary material for: A quasi-integral controller for adaptation of genetic modules to variable ribosome demand
Source: Nat Commun. 2018 Dec 21;9:5415. doi: 10.1038/s41467-018-07899-z (PMC6303309; doi:10.1038/s41467-018-07899-z)
Supplement: Supplementary file 3 — Description of Additional Supplementary Files [file 41467_2018_7899_MOESM3_ESM.pdf]

Title: Supplementary Software (MATLAB Code.zip):

Description: MATLAB script files including the following items: a. ReadMe.txt: a brief description of MATLAB scripts in this folder b. Main_Figure_Huang_et_al.m: MATLAB code to generate all main text figures and Supplementary Figures 15 and 18 c. Supp_Fig_16.m: MATLAB code to generate Supplementary Figure 16 d. Supp_Fig_17.m: MATLAB code to generate Supplementary Figure 17 e. The following custom MATLAB functions used in the scripts above: sRNA_silencing.m, sRNA_feedback.m, sRNA_feedback_sat.m and ActCascade.m.
